# Brewing Quality of Hop Varieties Cultivated in Central Italy Based on Multivolatile Fingerprinting and Bitter Acid Content

**DOI:** 10.3390/foods9050541

**Published:** 2020-04-29

**Authors:** Massimo Mozzon, Roberta Foligni, Cinzia Mannozzi

**Affiliations:** Department of Agricultural, Food and Environmental Sciences, Università Politecnica delle Marche, Via Brecce Bianche 10, 60131 Ancona, Italy; m.mozzon@staff.univpm.it

**Keywords:** hop variety, *Humulus lupulus*, bitter substances, humulones, volatile components, SPME

## Abstract

The brewing value of hops is mainly affected by the content and composition of humulones (α-acids) and essential oil. Interest in hop plantations is increasing more and more in Italy, in parallel with the rising number of microbreweries and brewpubs, which are strongly oriented towards local production chains. In this context, a selection of 15 international hop varieties were grown, under the same conditions, in an experimental field in the Marche region, Central Italy, with the aim of assessing their suitability for beer production. A multivariate analysis approach to experimental data showed a high content of α- and β-acids and myrcene in the Centennial, Brewer’s Gold, Sterling, Cascade, Nugget, and Columbus varieties; a consistently lower percentages of humulones and a predominance of sesquiterpene hydrocarbons in the cultivars Mount Hood, Northern Brewer, Northdown, Galena, Willamette, and Fuggle; and a desirable high α-acids content and a sesquiterpene-type aroma in cultivars Chinook, Yeoman, and Hallertau. Further studies are needed to assess the environmental adaptability and the yield performance of hop plants in the pedoclimatic conditions of the Central Italy hills.

## 1. Introduction

The unfertilized female inflorescence of hop plant (*Humulus lupulus* L.) (hops or cones), used in the brewing process in various forms (e.g., whole cones, pellets, and extracts), changes the wort characteristics and provides bitterness, aroma, astringency, and fullness to the finished product. Moreover, hops act as a technological aid (clarifier), contribute to the microbiological stability of beer, and enhance the foam-building ability of beer and foam stability [1]. The young shoots that emerge from the plant in April and May are also used for food preparations, both fresh (salads, risotto, and omelets) and preserved. The vitamin E content of shoots [2] could be higher than other well-known vegetable sources of tocopherols [3] and has been recently highlighted as a nutritional peculiarity of this culinary delicacy.

The brewing value of hops is primarily attributed to a complex mixture of secondary metabolites constituting the yellow resinous powder secreted by the lupulin glands of cones [4]. The chemical composition of hops is strongly affected by variety, ripening stage, environmental factors, agronomic practices, and cultivation area [5]. It is well established that humulones (α-acids) are the most important precursors of bitter substances in finished beer, even if several bitter tasting products of β-acid transformation are generated during wort boiling [1,6]. In fact, beer makers classify hop varieties on the basis of α-acid content into bitter (>7%) and finishing/aromatic ones (<7%) [7]. The hop essential oil is also important to the brewer, as it contributes to the characteristic “hoppy” flavor of the beer, even if the wort boiling and the fermentation process result in the loss or transformation of a large portion of the original hops aroma. However, it is not fully understood how the hop variety, the amount and form (dried cones, pellets, and extracts) of hops, and the hopping method (early, late, or dry) could impact the volatile fraction of beers [8]. The essential oil is primarily made up of terpene hydrocarbons and their oxidized derivatives; the oil content of dry cones ranges from 0.5% to 3% [9]. The volatile profile has been recently proposed for the cultivar characterization [9,10,11].

Wild hop plants normally grow in the whole Italian peninsula. However, winemaking has relegated brewing to second place for a long time and hop plantations in Italy have mostly had an amateur character. Currently, new regulations concerning the production of hops and the management of craft breweries management are increasing the interest of farmers and entrepreneurs in these activities. The increasing number of microbreweries and brewpubs [12] suggests new development opportunities for the beer market. These new entities are increasingly interested in producing beers within a local production chain, from ingredients (hops, malt) to the final product. Italy is still far from meeting the estimated need for hops at 3500 t/year. A recent research project, funded by the Italian Ministry of Agricultural, Food and Forestry Policies and coordinated by the “Consiglio per la ricerca in agricoltura e l’analisi dell’economia agraria” (CREA) [13], showed that the surface invested in hop plantation is just over 50 ha and that the business plantations (size greater than 1000 m^2^) are mostly concentrated in the Emilia Romagna and Lazio regions. The same study highlighted that Apennine areas of Central Italy are very suitable for cultivating hops for brewing. The excellent potential that the sector could express for the revitalization of agriculture has also been highlighted, especially in the internal marginal areas of the country. However, very little data are currently available on the chemical characteristics of “Italian” hops [14], thus, leaving to empiricism the development of distinctive hop characters in beers by craft brewers.

In this context, a selection of 15 international hop varieties were grown, under the same conditions, in an experimental field in the Marche region of Central Italy. The volatile fingerprint and the α- and β-acid content were compared to the standard characteristics of the varieties, with the aim of assessing the suitability of hops cultivated in Central Italy for beer production.

## 2. Materials and Methods

### 2.1. Plant Material

Fifteen international hop cultivars, from USA, UK, and Germany (3 bittering, 4 aroma, and 8 dual purpose) were used for the experiment (Table 1). The trial was carried out at the experimental field of “La Contea” farm (Tavullia, Pesaro Urbino, Italy), located in the Montelabbate (Pesaro Urbino, Italy) municipality (43°48′ N, 12°45′ E, altitude 260 m a.s.l.). Five to seven rhizomes per cultivar were planted, in the spring of 2015, in the typical silt clay soil of the Marche region hills. Hop plants were grown on a standard trellis system 8 m high. The plants were spaced 2.8 m between rows and 1 m apart in the rows. Weed growth was periodically checked, and weeds were manually removed. Pests and pathogens were controlled by preventive spraying of copper products before flowering. A drip irrigation system provided water to the plants whenever necessary.

Cones from the 2018 production year were harvested separately for each variety, freeze-dried and stored at −20 °C, until the analysis. Three samples of each cultivar were used for chemical analyses.

### 2.2. Determination of α- and β-Acids

The extraction of bitter tasting precursors was carried out according to Stevens et al. [16]. Briefly, 0.5 g of ground dried cones were thoroughly mixed with methanol into a 10 mL volumetric flask. In order to complete the extraction process, the mixture was allowed to stay in the dark for 4 h. The supernatant was filtered using a 0.22 µm PTFE membrane (Thermo Scientific, Waltham, MA, USA) and directly injected in an Agilent (Santa Clara, CA, USA) 1100 Series HPLC system, equipped with a Zorbax Eclipse Plus C18 column (3.0 × 150 mm, 3.5 μm particle size) and a UV detector set at 330 nm. A gradient elution was performed using the following two-solvent system [17]: solvent A = 75% methanol, 24% water, and 1% phosphoric acid; solvent B = methanol and solvent B was increased from 30% to 55% in 10 min. The flow rate was 0.8 mL/min. All solvents were purchased at Sigma-Aldrich (St. Louis, MO, USA).

The International Calibration Extract 4 (ICE-4) (Labor Veritas AG, Zürich, Switzerland) was used to quantify the α- and β-acids. A set of three standard methanolic solutions of ICE-4 (0.4, 1.0, and 2.0 mg/mL) were used to make the calibration curves.

### 2.3. GC-MS Analysis of Volatile Components

Volatiles sampling was carried out by headspace solid phase microextraction (HS-SPME), according to the operative parameters described by Savini et al. [18]. Volatiles were analyzed by gas chromatography-mass spectrometry in a Varian 3900 GC coupled with a Saturn 2100T ion trap mass detector (Varian Analytical Instruments, Walnut Creek, CA, USA) and equipped with a fused silica capillary column ZB-5 (30 m length, 0.25 mm internal diameter, 0.25 μm film thickness; Phenomenex, Torrance, CA, USA). The injector was operating in splitless mode for 0.1 min at a constant temperature of 250 °C; oven temperature was increased from 40 °C to 220 °C at a rate of 6 °C/min, then held at the final temperature for 5 min; carrier gas (He) was set at a constant flow mode (1.0 mL/min); the ion trap and the transfer line were set at 200 °C and 220 °C, respectively. Experiments of both electronic impact fragmentation (EI, 70 eV) and chemical ionization (CI) (reagent gas, methanol) were carried out. Full scan MS data were acquired in the mass range of 31–250 amu.

Volatile compounds were identified by matching mass spectral data with those collected in the NIST/EPA/NIH Mass Spectral Library (Version 2.0a, built 1 July 2002; National Institute of Standards and Technology), and Kovats retention Indices (RIs) with those available in the public access database Pubchem [19]. A C8–C20 normal alkanes mixture (Sigma-Aldrich, St. Louis, MO, USA) was used to calculate RIs in the experimental conditions. An automated spreadsheet [20] was used to simplify the calculation of RIs of the unknown components and speeding up the comparison with the published indices. The CI spectral data (parent and base peaks) were used to confirm the molecular weight of volatile substances.

### 2.4. Data Analysis

Multivariate data analyses were carried out to explore the structure of the experimental data (principal component analysis, PCA) and to classify varieties (cluster analysis). A Pearson’s correlation analysis was also carried out to assess the relationships among experimental variables. The Tukey–Kramer’s honest significant difference (HSD) test was used to compare the experimental variables among varieties. All statistical analyses were carried out by the software JMP^®^ Version 10 (SAS Institute Inc., Cary, NC, USA).

## 3. Results and Discussion

### 3.1. Precursors of Bitter Tasting Compounds of Beers

Table 2 reports the acid contents of hop cones harvested from the 15 varieties. It is well established that most of the perceived pleasant bitterness in beer is provided by isomerization derivatives of α-acids and that isohumulones are more bitter than isocohumulones [1]. 

The highest levels of α-acids were observed in Nugget (NUG) (10.61% ± 0.20% DM), Chinook (CHI) (7.98% ± 0.37% DM), and Yeoman (YEO) (7.81% ± 0.03% DM), while Cascade (CAS), NUG, and Mount Hood (MHO) had the highest contents of lupulones (7.39% ± 1.42%, 4.38% ± 0.11%, and 4.26% ± 0.17% DM, respectively). However, the amount of bitter precursors of the cultivars analyzed was generally lower than the typical value of the corresponding commercial varieties (Table 1), with the exception of CAS and NUG. As reported above, a low contribution of cohumulone to the α-acid fraction is also desirable. The cohumulone percentage (of total α-acids) of most of the analyzed samples lay in the range of the reference values in Table 1, while Willamette (WIL) (47.7%), Fuggle (FUG) (32.5%), and Sterling (STE) (35.3%) were characterized by a more unfavorable composition of the α-acid fraction. Cohumulone percentages lower than commercial cultivars were observed in Galena (GAL) (29.3%), Brewer’s Gold (BRG) (36.6%), CAS (30.6%), and CHI (26.8%).

Mongelli et al. [14] reported higher percentages of α- and β-acids for the hop varieties Columbus (COL) (19.6%, 7.2%), FUG (4.5%, 6.5%), and WIL (2.9%, 2.1%) cultivated in Northern Italy, thus, confirming the strong influence of pedoclimatic conditions on hops chemical traits. Pearson et al. [21] reported similar results for COL and CHI varieties cultivated in an open-sided greenhouse in Central Florida, which were characterized by an α-acid content of 4.8% to 6.8% and 9.7% to 10.4% and a β-acid content of 2.7% to 2.8% and 2.1% to 2.5%, respectively. Lafontaine et al. [6] observed in CAS cones collected from 5 to 6 weeks over the years 2014 to 2016 a rough maintenance of the humulones (4.40% to 5.79%) and lupulones (5.81% to 8.50%) concentration throughout the overall harvest period. Conversely, Drexler et al. [22] showed the importance of the harvest time for Hallertauer Mittelfrüh hops; a significant increase of the lupulones concentration from the first to the fourth week or inflorescence development was observed.

### 3.2. Aroma Components of Cones

A total of 57 volatile substances (19 sesquiterpene hydrocarbons, 15 monoterpene hydrocarbons, 13 esters, four ketones, three oxygenated sesquiterpenes, two *n*-alkanes, one oxygenated monoterpene) were positively or tentatively identified, on the basis of the criteria described in Section 2.3. (Table 3, Supplementary Figures S1 and S2). Hydrocarbons accounted for 90% to 98% of the total volatile components. Sesquiterpenes (hydrocarbons and oxygenated analogues) were the most abundant chemical class in Hallertau (HAL), WIL, YEO, FUG, MHO, Northern Brewer (NBR), GAL, and Northdown (NOR), while monoterpene hydrocarbons were the most represented aroma components in Centennial (CEN), BRG, STE, CAS, NUG, COL, and CHI.

Myrcene, humulene, and caryophyllene were the major sesquiterpenoids in all samples. Myrcene is mainly responsible for the characteristic pungent smell of fresh hops, while humulene and caryophyllene are recognized to be precursors of oxygenated aroma-active substances during wort boiling, thus, providing the spicy/herbal hop character to beer [11]. Significant differences were observed between the aroma composition of the experimental samples and the commercial varieties (Table 4). The relative levels of caryophyllene in the experimental hops WIL, YEO, CEN, FUG, MHO, GAL, BRG, and STE where higher than those found in the commercial cultivars. Higher relative percentages of humulene were also observed in the experimental YEO, FUG, GAL, and BRG, together with a lower level of myrcene. In addition, CAS, NUG, COL, NOR, and CHI hops from the Marche region plantation were richer in myrcene than commercial plants. Mongelli et al. [14] reported comparable values for humulene and caryophyllene percentages of COL, FUG, and WIL cultivated in Northern Italy, while the myrcene contents of FUG and WIL were very low (0.91% and 0.83%, respectively).

Other terpenic hydrocarbons were distinctive of some hop cultivars. WIL was previously reported to be a high-farnesene hop cultivar [23]; however, YEO and CHI cultivated in the Marche hills showed higher percentages of farnesene isomers. A group of experimental cultivars of American origin (CEN, STE, CAS, NUG, and COL), together with BRG (UK), showed the highest β-pinene percentages (1.67% to 2.26%). The interest concerning this monoterpene lies in its role in the initiation of autoxidation of α-acids [24]. Amounts of β-phellandrene higher than 1% were only found in CEN, BRG, CAS, and COL. (E)-β-ocimene contents were much lower than 1% except in NBR, BRG, NUG, and COL (1.01% to 1.39%). (E)-calamenene and α-calacorene characterized the aroma of HAL (1.45% and 1.30%, respectively) and CHI (0.90% and 0.66%, respectively), while selinene isomers characterized YEO volatile fraction (7.93%). The percentages of α- and β-selinene observed in COL, FUG, and WIL were similar to those reported by Mongelli et al. [14] for the same varieties cultivated in Northern Italy.

Esters and ketones were relatively minor groups, ranging from 0.66% (WIL) to 9.24% (COL) and from 0.15% (WIL) to 2.09% (GAL) of the total volatiles, respectively. Esters are known for their fruity notes [11]. According to Yan et al. [10], the most abundant ketone was 2-undecanone (ranging from 0.01% in BRG to 1.22% in GAL), while isoamyl isobutyrate was the most abundant ester. COL was the richest cultivar (4.32%); in YEO, BRG, NUG, and CHI varieties the isoamyl isobutyrate contents were in the range 1.16% to 2.36%; in all the other varieties the quantitative contribution of this ketone to aroma was much lower than 1%.

Among the hop oil components, linalool is widely accepted as being flavor-active in beer and it is associated with floral impressions in both hops and beer [25]. In fact, terpenic alcohols (linalool and geraniol) were detected as predominant compounds in the pitching wort and in late and dry hopped beers, followed by monoterpene (β-myrcene) and sesquiterpene hydrocarbons (humulene and β-caryophyllene). Differences in polarity, volatility, and solubility were used to explain the more selective retention of alcohols than hydrocarbons [8]. The linalool content of the experimental cultivars in this study ranged from 0.17% (YEO) to 0.94% (HAL).

### 3.3. Multivariate Analysis

**Figure 1:**
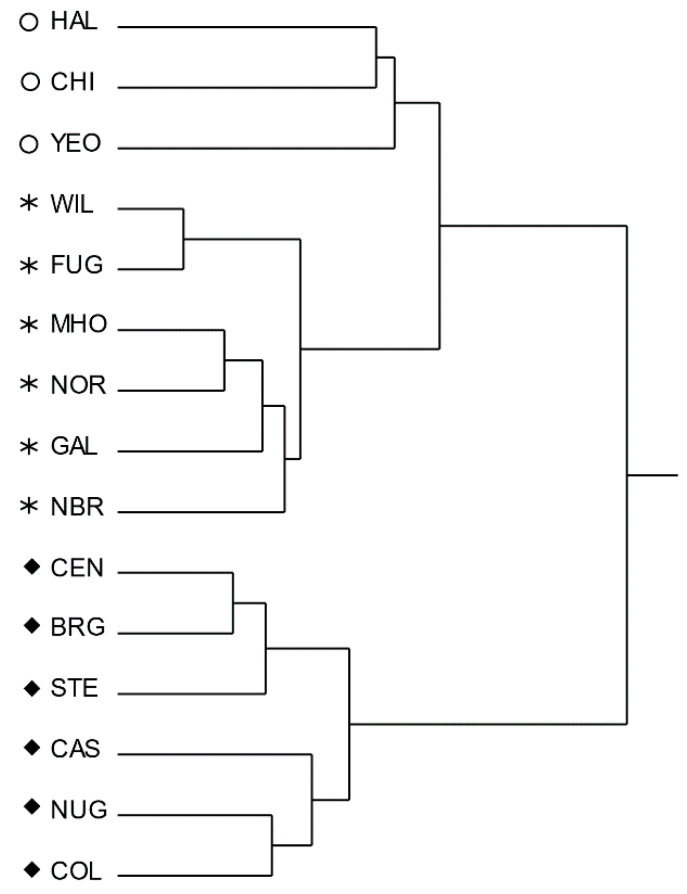
Dendrogram of the hierarchical cluster analysis among commercial varieties of *H. lupulus* L. cultivated in the Marche region, as determined by chemical similarity (Ward, distance scale).

Hierarchical clustering split samples into three groups, on the basis of their volatile profiles (18 variables with at least one value greater than 1%) and the vinylogous type of organic acids (four variables) (Figure 1).

The Group A cultivars (CEN, BRG, STE, CAS, NUG, COL) had both the highest levels of humulones (2.13% to 10.61%) and lupulones (1.79% to 7.39%) and the highest ratio between mono- (myrcene) and sesquiterpene (humulene + caryophyllene) hydrocarbons. The Group B varieties (WIL, FUG, MHO, NOR, GAL, and NBR) had the lowest contents of α-acids (0.65% to 3.44%) and their aroma showed consistently higher percentages of the sesquiterpenes humulene (25.63% to 46.10%) and caryophyllene (18.17% to 25.54%). Group C (HAL, CHI, YEO) showed intermediate characteristics. The grouping did not fully reflect the general characteristics of the commercial varieties in Table 1, according to their brewing use, maturity timelines, origin, and chemical parameters. However, most of aroma hops (WIL, FUG, and MHO) belonged to Group B and two out of three bittering hops belonged to Group A. The dual purpose hops were mainly included in Group A as well. Rossini et al. [26] reported good yield performance for the cultivars CAS and YEO under the climatic conditions of Central Italy, and a more complex appreciated profile for beers flavored with local cones than those hopped with commercial products. Our experimental data also showed interesting properties for those two varieties, i.e., a content of α-acid comparable to commercial variety, a favorable composition of the humulones fraction for CAS, and a high level of α-acids and sesquiterpene hydrocarbons for YEO.

**Figure 2:**
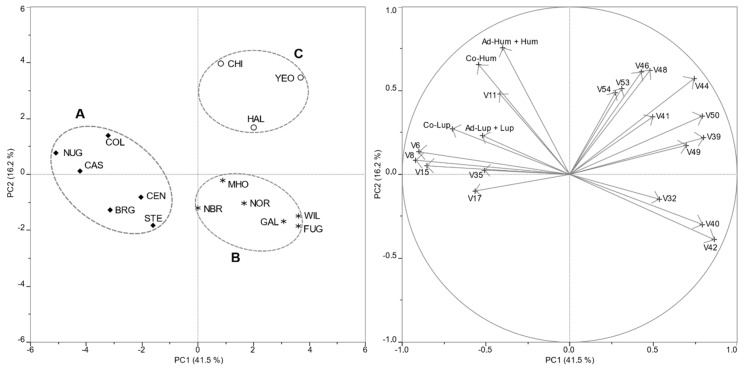
(**Left**) PCA scores plot of chemical data of hop cones from 15 commercial varieties of *H. lupulus* L. cultivated in the Marche region. Cultivar identifiers are as in Table 1; (**Right**) PCA loadings plot of variables (volatiles and acids). Identifiers of chemical substances are as in Table 1 and Table 2.

The PCA reflected these relationships (Figure 2). The first two principal components explained 57.7% of the total variance. PC1 was mainly affected by a cluster of monoterpene hydrocarbons (myrcene, β-pinene, and β-phellandrene) with negative loadings, and a cluster of sesquiterpene hydrocarbons (humulene, caryophyllene, copaene, muurolene, farnesene, and selinene) with positive loadings. Sesquiterpene hydrocarbons (humulene, caryophyllene) had negative loadings on PC2, whereas humulones only affected PC2. Positive linear correlations were found between β-pinene and β-phellandrene (*r* = 0.9471) and between the pairs of sesquiterpenes caryophyllene vs. humulene (*r* = 0.9149), β-selinene vs. α-selinene (*r* = 0.9916), γ-muurolene vs. α-cadinene (*r* = 0.9421, and (E)-calamenene vs. α-calacorene (*r* = 0.0.9945). Humulones were also positively correlated (*r* = 0.9619), as well as lupulones (*r* = 0.9022), while no correlations (variables positioned in approximately orthogonal directions between them) were found between the aroma components and the precursors of bitter tasting compounds. The strongest inverse correlations (variables positioned in opposite direction respect to the axes origin and far from the plot origin) were found between myrcene and the sesquiterpenes caryophyllene (*r* = −0.9276) and humulene (*r* = −0.9318).

PC1 was able to differentiate the cultivars that fell into Group A (highest myrcene percentages and lowest humulene and caryophyllene relative amounts) from the others. Lower contents of humulones and higher relative amounts of sesquiterpenes (humulene and caryophyllene) drove the differentiation between cultivars that fell into Groups B and C toward the lower right and the upper right quadrant, respectively.

## 4. Conclusions

The hop plant can adapt to different environments and pedoclimatic conditions, because of its rustic habit, high degree of intraspecific genetic variability, and easiness of cultivation. However, difficulties in plantation management (high trellis systems, long periods of time before plants are ready for data collection, manual harvesting of cones) have hindered systematic studies on environmental adaptability and yield performance of hop cultivars in different countries.

The present study aimed to identify, among a selection of 15 commercial hop varieties of different origin and traditional use, those potentially suited to the Central Italy hilly environment in terms of brewing quality. Multivariate analysis of experimental data concerning the volatiles and acid profiles identified a group of cultivars (CEN, BRG, STE, CAS, NUG, and COL) characterized by high contents of α- and β-acids and a prevalence of monoterpenes (namely myrcene) in their aroma. Precursors of the spicy/herbal hop character of beer (the sesquiterpenes humulene and caryophyllene) predominated in all the other varieties. Particularly, the cultivars MHO, NBR, NOR, GAL, WIL, and FUG showed consistently lower percentages of humulones, while the varieties CHI, YEO, and HAL had a desirable high α-acids content and a sesquiterpene-type aroma. CHI and YEO appeared to be the most promising varieties, due to the highest level of α-acids (CHI and YEO), the most favorable composition of the humulone fraction (CHI), and the highest percentage of volatile precursors of the hoppy character of beer (YEO).

The comparison, with very little available data concerning chemical and agronomical traits of hop varieties cultivated in different Italian regions, highlighted the strong influence of pedoclimatic conditions on the overall brewing quality of cones, and thus limited the applicability of experimental data to different environments. These results suggest the need to further investigate the environmental adaptability and the agronomic performance of hop varieties in the pedoclimatic conditions of the hills of Central Italy. Experimental data should be collected for more years, as most of the Italian hop plantations are relatively young, and therefore still not qualitatively stable. Additionally, the extreme variability of plantation management and post-harvesting processing (drying and storing) of cones add difficulty to data comparison.

## Figures and Tables

**Table 1:** List of hop varieties used for the experiment, their brewing use, maturity timelines, origin, and chemical characters [15].

Sample ID	Name	Brewing Use	Seasonal Maturity ^1^	Origin	*α*-Acids% ^2^	*β*-Acids% ^2^	Cohumulone%	Myrcene%	Humulene%	Caryophyllene%
HAL	Hallertau	Aroma	E to M	Germany	3.0–3.5	3.5–4.5	20–26	35–44	30–55	10–15
WIL	Willamette	Aroma	E to M	USA	4–6	3–4	30–35	30–55	20–30	7–8
YEO	Yeoman	Dual purpose	E	UK	12–16	4–5	25	48	20	10
CEN	Centennial	Dual purpose	M	USA	9.5–11.5	3.5–4.5	28–30	45–55	10–18	5–8
FUG	Fuggle	Aroma	E to M	UK	2.4–6.1	2.1–2.8	25–29	43.4	26.6	9.1
MHO	Mount Hood	Aroma	E to M	USA	4–8	5–8	21–23	30–40	12–38	7–16
NBR	Northern Brewer	Dual purpose	E to M	Germany	7–10	3.5–5	27–33	25–45	35–50	10–20
GAL	Galena	Bittering	M	USA	12	7.5	39	55–60	10–15	3–6
BRG	Brewer’s Gold	Bittering	L	UK	7.1–11.3	3.3–6.1	41	66.7	11.6	6.5
STE	Sterling	Dual purpose	M	USA	4.5–9	4–6	21–28	44–48	19–23	5–8
CAS	Cascade	Dual purpose	M	USA	4.5–8.9	3.6–7.5	33–40	45–60	8–16	4–6
NUG	Nugget	Bittering	M	USA	9.5–14	4.2–5.8	22–30	48–59	12–22	7–10
COL	Columbus	Dual purpose	M to L	USA	14–18	4.5–6	28–35	25–55	9–25	6–12
NOR	Northdown	Dual purpose	M	UK	7–10	4–5.5	24–32	23–29	37–45	13–17
CHI	Chinook	Dual purpose	M to L	USA	12–14	3–4	29–34	35–40	18–25	9–11

^1^ M, medium; L, late; E, early. ^2^ On dry matter basis.

**Table 2:** Content of *α*- and *β*-acids (g/100 g dry matter, mean ± SD) of hop cones harvested from 15 varieties cultivated in the Marche region (Central Italy).

Sample ID	CoH	AdH + Hum	CoL	AdL + Lup	Total *α*-Acids	Total *β*-Acids
HAL	0.34 ± 0.05 ^g^	1.24 ± 0.20 ^efg^	0.76 ± 0.13 ^efg^	1.23 ± 0.20 ^cdef^	1.58 ± 0.24 ^ef^	1.99 ± 0.33 ^de^
WIL	0.31 ± 0.03 ^g^	0.34 ± 0.00 ^g^	1.25 ± 0.01 ^def^	1.09 ± 0.01 ^def^	0.65 ± 0.03 ^f^	2.34 ± 0.02 ^de^
YEO	1.86 ± 0.00 ^cd^	5.94 ± 0.03 ^b^	1.34 ± 0.01 ^cde^	1.66 ± 0.00 ^bcd^	7.81 ± 0.03 ^b^	3.00 ± 0.01 ^bcd^
CEN	0.72 ± 0.08 ^fg^	1.80 ± 0.22d ^ef^	1.08 ± 0.14 ^defg^	1.30 ± 0.18 ^cdef^	2.51 ± 0.29 ^de^	2.37 ± 0.32 ^de^
FUG	0.40 ± 0.03 ^g^	0.83 ± 0.10f ^g^	0.46 ± 0.06 ^fg^	0.42 ± 0.07 ^f^	1.23 ± 0.13 ^ef^	0.88 ± 0.13 ^e^
MHO	0.77 ± 0.04 ^fg^	2.51 ± 0.11 ^cd^	1.77 ± 0.08 ^bcd^	2.49 ± 0.09 ^b^	3.28 ± 0.15 ^cd^	4.26 ± 0.17 ^bc^
NBR	1.08 ± 0.13 ^ef^	2.36 ± 0.30 ^cde^	1.21 ± 0.12 ^defg^	1.14 ± 0.10 ^def^	3.44 ± 0.42 ^cd^	2.35 ± 0.22 ^de^
GAL	0.29 ± 0.11 ^g^	0.70 ± 0.26 ^fg^	0.41 ± 0.17 ^g^	0.53 ± 0.21 ^ef^	0.99 ± 0.37 ^ef^	0.95 ± 0.38 ^e^
BRG	0.78 ± 0.06 ^fg^	1.35 ± 0.11d ^efg^	1.06 ± 0.10 ^defg^	0.74 ± 0.07 ^def^	2.13 ± 0.17 ^def^	1.79 ± 0.17 ^de^
STE	0.79 ± 0.09 ^fg^	1.45 ± 0.02d ^efg^	1.36 ± 0.10 ^cde^	1.68 ± 0.11 ^bcd^	2.24 ± 0.11 ^def^	3.04 ± 0.21 ^bcd^
CAS	1.37 ± 0.24 ^de^	3.10 ± 0.65 ^c^	3.14 ± 0.61 ^a^	4.25 ± 0.81 ^a^	4.47 ± 0.89 ^c^	7.39 ± 1.42 ^a^
NUG	2.90 ± 0.05 ^a^	7.71 ± 0.15 ^a^	2.23 ± 0.06 ^b^	2.15 ± 0.06 ^bc^	10.61 ± 0.20 ^a^	4.38 ± 0.11 ^b^
COL	2.49 ± 0.35 ^ab^	4.92 ± 0.70 ^b^	2.10 ± 0.33 ^bc^	1.44 ± 0.23 ^cde^	7.41 ± 1.05 ^b^	3.54 ± 0.57 ^bcd^
NOR	0.63 ± 0.08 ^fg^	1.52 ± 0.19 ^defg^	0.87 ± 0.13 ^efg^	0.99 ± 0.14 ^def^	2.15 ± 0.28 ^def^	1.86 ± 0.26 ^de^
CHI	2.14 ± 0.10 ^bc^	5.84 ± 0.28 ^b^	1.29 ± 0.06 ^de^	1.28 ± 0.06 ^cdef^	7.98 ± 0.37 ^b^	2.56 ± 0.11 ^cde^

CoH, cohumulone; AdH, adhumulone; Hum, humulone; CoL, colupulone; AdL, adlupulone; Lup, lupulone. Values in a column with different letters are significantly different (Tukey test, *p* < 0.05).

**Table 3:** Volatile components identified in the headspace of dried hop cones harvested from 15 varieties cultivated in the Marche region (Central Italy).

Peak ID	RT (min)	RI	CI Parent and Base Ions	Name	CAS Number	Category
V1	6.616	919	145 [M + 1]	isobutyl isobutyrate	97-85-8	Ester
V2	6.773	926	137 [M + 1]	tricyclene	508-32-7	Monoterpene hydrocarbon
V3	6.947	934	137 [M + 1]	α-thujene	2867-05-2	Monoterpene hydrocarbon
V4	7.119	942	137 [M + 1]	α-pinene	80-56-8	Monoterpene hydrocarbon
V5	7.472	957	137 [M + 1]	camphene	79-92-5	Monoterpene hydrocarbon
V6	8.147	983	137 [M + 1]	β-pinene	127-91-3	Monoterpene hydrocarbon
V7	8.356	991	127 [M + 1]. 109 [M + 1 − H_2_O]	6-methyl-5-heptene-2-one	110-93-0	Ketone
V8	8.510	996	137 [M + 1]	β-myrcene	123-35-3	Monoterpene hydrocarbon
V9	8.810	1009	137 [M + 1]	α-phellandrene	99-83-2	Monoterpene hydrocarbon
V10	8.957	1015	158 [M]	2-methylbutyl isobutyrate	2445-69-4	Ester
V11	9.044	1019	158 [M]	isoamyl isobutyrate	2050-01-3	Ester
V12	9.105	1022	137 [M + 1]	α-terpinene	99-86-5	Monoterpene hydrocarbon
V13	9.251	1028	145 [M + 1]	methyl heptanoate	106-73-0	Ester
V14	9.332	1032	143 [M + 1]	heptenoic acid isomer. methyl ester		Ester
V15	9.413	1035	137 [M + 1]	β-phellandrene	555-10-2	Monoterpene hydrocarbon
V16	9.594	1043	137 [M + 1]	(Z)-β-ocimene	3338-55-4	Monoterpene hydrocarbon
V17	9.859	1054	137 [M + 1]	(E)-β-ocimene	3779-61-1	Monoterpene hydrocarbon
V18	10.147	1065	137 [M + 1]	y-terpinene	99-85-4	Monoterpene hydrocarbon
V19	10.203	1067	159 [M + 1]	heptanoic acid. 2-methyl. methyl ester	51209-78-0	Ester
V20	10.808	1090	159 [M + 1]	heptanoic acid. 6-methyl. methyl ester	2519-37-1	Ester
V21	10.883	1092	137 [M + 1]	terpinolene	586-62-9	Monoterpene hydrocarbon
V22	10.931	1094	143 [M + 1]	2-nonanone	821-55-6	Ketone
V23	11.152	1102	137 [M + 1 − H_2_O]	linalool	78-70-6	Oxygenated monoterpene
V24	11.726	1128	159 [M + 1]	methyl octanoate	111-11-5	Ester
V25	11.862	1134	137 [M + 1]	alloocimene	7216-56-0	Monoterpene hydrocarbon
V26	12.173	1147	137 [M + 1]	Unidentified		Monoterpene hydrocarbon
V27	13.390	1195	157 [M + 1]	2-decanone	693-54-9	Ketone
V28	13.549	1201	157 [M + 1 − CH_2_]	n-dodecane	112-40-3	*n*-alkane
V29	13.846	1215	171 [M + 1]. 139 [M + 1 − CH_3_OH]	methyl x-nonenoate		Ester
V30	14.129	1227	173 [M + 1]	methyl nonanoate	1731-84-6	Ester
V31	14.638	1250	187 [M + 1]	heptyl isobutanoate	2349-13-5	Ester
V32	15.749	1295	171 [M + 1]	2-undecanone	112-12-9	Ketone
V33	15.874	1300	171 [M + 1 − CH_2_]	n-tridecane	629-50-5	*n*-alkane
V34	16.092	1311	185 [M + 1]. 153 [M + 1 − CH_3_OH]	methyl 4-decenoate	1191-02-2	Ester
V35	16.443	1328	187 [M + 1]	methyl decanoate	110-42-9	Ester
V36	17.100	1358	205 [M + 1]	α-cubebene	17699-14-8	Sesquiterpene hydrocarbon
V37	17.208	1362	205 [M + 1]	sativene	3650-28-0	Sesquiterpene hydrocarbon
V38	17.610	1380	205 [M + 1]	ylangene	14912-44-8	Sesquiterpene hydrocarbon
V39	17.711	1384	205 [M + 1]	α-copaene	3856-25-5	Sesquiterpene hydrocarbon
V40	18.717	1432	205 [M + 1]	b-caryophillene	87-44-5	Sesquiterpene hydrocarbon
V41	18.879	1440	205 [M + 1]	β-cubebene	13744-15-5	Sesquiterpene hydrocarbon
V42	19.463	1468	205 [M + 1]	humulene	6753-98-6	Sesquiterpene hydrocarbon
V43	19.787	1483	205 [M + 1]	b-copaene	18612-33-4	Sesquiterpene hydrocarbon
V44	19.844	1486	205 [M + 1]	(E)-b-Famesene	18794-84-8	Sesquiterpene hydrocarbon
V45	19.916	1489	205 [M + 1]	α-Farnesene	502-61-4	Sesquiterpene hydrocarbon
V46	20.095	1497	205 [M + 1]	b-selinene	17066-67-0	Sesquiterpene hydrocarbon
V47	20.212	1503	205 [M + 1]	α-muurolene	31983-22-9	Sesquiterpene hydrocarbon
V48	20.276	1506	205 [M + 1]	α-selinene	473-13-2	Sesquiterpene hydrocarbon
V49	20.626	1524	205 [M + 1]	γ-muurolene	30021-74-0	Sesquiterpene hydrocarbon
V50	20.784	1532	205 [M + 1]	α-cadinene	11044-40-9	Sesquiterpene hydrocarbon
V51	20.844	1535	205 [M + 1]	b-cadinene	523-47-7	Sesquiterpene hydrocarbon
V52	20.986	1543	205 [M + 1]	cadina-1.4-diene	16728-99-7	Sesquiterpene hydrocarbon
V53	21.090	1548	205 [M + 1]	(E)-calamenene	40772-39-2	Sesquiterpene hydrocarbon
V54	21.210	1554	205 [M + 1]	α-calacorene	21391-99-1	Sesquiterpene hydrocarbon
V55	22.057	1595	203 [M + 1 − H_2_O]	caryophillene oxyde	1139-30-6	Oxygenated sesquiterpene
V56	22.356		203 [M + 1 − H_2_O]	Unidentified		Oxygenated sesquiterpene
V57	22.567		203 [M + 1 − H_2_O]	Unidentified		Oxygenated sesquiterpene

RT, retention time; RI, Kovats retention index (DB-5 equivalent column); CI, chemical ionization experiment.

**Table 4:** Aroma composition (chromatographic area %, mean ± SD) of hop cones harvested from 15 varieties cultivated in the Marche region (Central Italy).

Peak ID	HAL	WIL	YEO	CEN	FUG	MHO	NBR	GAL	BRG	STE	CAS	NUG	COL	NOR	CHI
V1	0.03 ± 0.00 ^b^	0.03 ± 0.00 ^b^	0.28 ± 0.18 ^b^	0.08 ± 0.06 ^b^	0.05 ± 0.04 ^b^	0.05 ± 0.02 ^b^	0.09 ± 0.03 ^b^	0.08 ± 0.08 ^b^	0.35 ± 0.10 ^b^	0.04 ± 0.00 ^b^	0.11 ± 0.04 ^b^	0.44 ± 0.09 ^a^	0.90 ± 0.42 ^a^	0.07 ± 0.03 ^b^	0.30 ± 0.02 ^b^
V2	0.01 ± 0.00	0.00 ± 0.00	0.00 ± 0.00	0.01 ± 0.01	0.02 ± 0.01	0.01 ± 0.00	0.01 ± 0.00	0.00 ± 0.00	0.01 ± 0.00	0.00 ± 0.00	0.01 ± 0.01	0.00 ± 0.00	0.07 ± 0.09	0.01 ± 0.00	0.01 ± 0.00
V3	0.02 ± 0.00 ^ab^	0.00 ± 0.00 ^b^	0.00 ± 0.00 ^b^	0.04 ± 0.03 ^ab^	0.14 ± 0.12 ^a^	0.01 ± 0.00 ^ab^	0.01 ± 0.00 ^ab^	0.00 ± 0.00 ^b^	0.02 ± 0.01 ^ab^	0.02 ± 0.00 ^ab^	0.03 ± 0.01 ^ab^	0.04 ± 0.02 ^ab^	0.05 ± 0.02 ^ab^	0.01 ± 0.00 ^b^	0.01 ± 0.00 ^ab^
V4	0.08 ± 0.03	0.07 ± 0.03	0.15 ± 0.06	0.35 ± 0.35	0.09 ± 0.06	0.12 ± 0.06	0.18 ± 0.03	0.06 ± 0.04	0.22 ± 0.04	0.24 ± 0.07	0.25 ± 0.14	0.24 ± 0.04	0.31 ± 0.10	0.19 ± 0.12	0.22 ± 0.00
V5	0.00 ± 0.00 ^c^	0.00 ± 0.00 ^bc^	0.02 ± 0.01 ^abc^	0.02 ± 0.02 ^abc^	0.01 ± 0.00 ^bc^	0.00 ± 0.00 ^c^	0.01 ± 0.00 ^bc^	0.01 ± 0.00 ^bc^	0.02 ± 0.01 ^abc^	0.01 ± 0.00 ^abc^	0.04 ± 0.03 ^abc^	0.06 ± 0.01 ^a^	0.06 ± 0.02 ^ab^	0.02 ± 0.01 ^abc^	0.02 ± 0.01 ^abc^
V6	0.85 ± 0.21	0.44 ± 0.17	0.57 ± 0.24	2.49 ± 2.19	0.50 ± 0.35	1.03 ± 0.42	0.92 ± 0.17	0.49 ± 0.25	1.81 ± 0.20	1.67 ± 0.18	1.93 ± 0.86	2.00 ± 0.33	2.26 ± 0.74	1.01 ± 0.48	1.60 ± 0.11
V7	0.01 ± 0.01 ^ab^	0.02 ± 0.01 ^ab^	0.11 ± 0.09 ^ab^	0.00 ± 0.00 ^b^	0.06 ± 0.03 ^ab^	0.01 ± 0.00 ^ab^	0.05 ± 0.03 ^ab^	0.04 ± 0.00 ^ab^	0.01 ± 0.01 ^ab^	0.02 ± 0.00 ^ab^	0.01 ± 0.01 ^ab^	0.04 ± 0.01 ^ab^	0.12 ± 0.02 ^a^	0.04 ± 0.01 ^ab^	0.00 ± 0.00 ^b^
V8	41.44 ± 1.94 ^bcd^	14.14 ± 0.04 ^e^	14.95 ± 6.07 ^e^	49.45 ± 4.05 ^abc^	14.08 ± 2.47 ^e^	40.82 ± 7.23 ^bcd^	34.01 ± 2.05 ^cd^	26.33 ± 3.53 ^b^	54.05 ± 5.40	53.69 ± 5.85 ^ab^	67.10 ± 5.66 ^a^	64.45 ± 1.34 ^a^	45.20 ± 4.84 ^bc^	33.72 ± 4.43 ^cd^	45.35 ± 5.24 ^bc^
V9	0.01 ± 0.00 ^b^	0.01 ± 0.00 ^b^	0.06 ± 0.05 ^ab^	0.01 ± 0.01 ^b^	0.05 ± 0.04 ^b^	0.02 ± 0.01 ^b^	0.03 ± 0.00 ^b^	0.03 ± 0.02 ^b^	0.04 ± 0.01 ^b^	0.02 ± 0.00 ^b^	0.01 ± 0.01 ^b^	0.03 ± 0.00 ^b^	0.12 ± 0.01 ^a^	0.04 ± 0.01 ^b^	0.04 ± 0.00 ^b^
V10	0.01 ± 0.00	0.01 ± 0.01	0.37 ± 0.22	0.05 ± 0.04	0.07 ± 0.05	0.02 ± 0.01	0.10 ± 0.03	0.12 ± 0.12	0.04 ± 0.03	0.01 ± 0.00	0.08 ± 0.08	0.21 ± 0.26	0.10 ± 0.12	0.09 ± 0.04	0.33 ± 0.02
V11	0.03 ± 0.03 ^d^	0.15 ± 0.04 ^d^	1.16 ± 0.62 ^bcd^	0.28 ± 0.12 ^cd^	0.18 ± 0.18 ^d^	0.11 ± 0.03 ^d^	0.28 ± 0.11 ^cd^	0.60 ± 0.84 ^bcd^	2.16 ± 0.52 ^bc^	0.03 ± 0.03 ^d^	0.20 ± 0.14 ^d^	1.85 ± 0.01 ^bcd^	4.32 ± 1.41 ^a^	0.30 ± 0.08 ^cd^	2.36 ± 0.07 ^b^
V12	0.01 ± 0.00	0.01 ± 0.01	0.23 ± 0.26	0.04 ± 0.04	0.47 ± 0.50	0.02 ± 0.02	0.04 ± 0.01	0.01 ± 0.01	0.06 ± 0.00	0.03 ± 0.00	0.20 ± 0.23	0.02 ± 0.01	0.06 ± 0.01	0.03 ± 0.01	0.06 ± 0.01
V13	0.14 ± 0.05 ^bc^	0.01 ± 0.01 ^c^	0.13 ± 0.06 ^bc^	0.12 ± 0.05 ^bc^	0.07 ± 0.04 ^bc^	0.23 ± 0.07 ^abc^	0.23 ± 0.19 ^abc^	0.13 ± 0.14 ^bc^	0.35 ± 0.09 ^ab^	0.13 ± 0.00 ^bc^	0.17 ± 0.02 ^bc^	0.50 ± 0.06 ^a^	0.23 ± 0.05 ^abc^	0.09 ± 0.05 ^bc^	0.27 ± 0.06 ^abc^
V14	0.62 ± 0.07	0.07 ± 0.00	0.06 ± 0.03	0.20 ± 0.12	0.15 ± 0.05	0.20 ± 0.05	0.29 ± 0.22	0.16 ± 0.12	0.06 ± 0.07	0.16 ± 0.02	0.11 ± 0.03	0.25 ± 0.02	0.44 ± 0.46	0.19 ± 0.05	0.11 ± 0.02
V15	0.68 ± 0.05 ^ab^	0.30 ± 0.02 ^b^	0.44 ± 0.18 ^ab^	1.46 ± 0.79 ^a^	0.41 ± 0.18 ^ab^	0.73 ± 0.26 ^ab^	0.87 ± 0.16 ^ab^	0.42 ± 0.10 ^ab^	1.22 ± 0.32 ^ab^	0.99 ± 0.01 ^ab^	1.08 ± 0.03 ^ab^	0.95 ± 0.02 ^ab^	1.16 ± 0.39 ^ab^	0.60 ± 0.19 ^bc^	0.92 ± 0.04 ^ab^
V16	0.02 ± 0.00 ^b^	0.02 ± 0.00 ^b^	0.05 ± 0.03 ^b^	0.05 ± 0.05 ^b^	0.03 ± 0.00 ^b^	0.02 ± 0.01 ^b^	0.13 ± 0.02 ^a^	0.01 ± 0.01 ^b^	0.04 ± 0.01 ^b^	0.04 ± 0.01 ^b^	0.03 ± 0.01 ^b^	0.04 ± 0.00 ^b^	0.01 ± 0.01 ^b^	0.05 ± 0.01 ^b^	0.02 ± 0.00 ^b^
V17	0.03 ± 0.00 ^b^	0.13 ± 0.05 ^b^	0.09 ± 0.06 ^b^	0.14 ± 0.10 ^b^	0.09 ± 0.03 ^b^	0.06 ± 0.02 ^b^	1.39 ± 0.21 ^a^	0.10 ± 0.08 ^b^	1.17 ± 0.41 ^a^	0.07 ± 0.01 ^b^	0.23 ± 0.02 ^b^	1.01 ± 0.09 ^a^	1.09 ± 0.27 ^a^	0.26 ± 0.01 ^b^	0.06 ± 0.01 ^b^
V18	0.02 ± 0.01	0.02 ± 0.00	0.02 ± 0.00	0.06 ± 0.02	0.04 ± 0.04	0.03 ± 0.01	0.14 ± 0.16	0.01 ± 0.00	0.04 ± 0.01	0.02 ± 0.00	0.04 ± 0.00	0.04 ± 0.01	0.10 ± 0.01	0.02 ± 0.01	0.04 ± 0.01
V19	0.15 ± 0.20	0.12 ± 0.04	0.01 ± 0.01	0.02 ± 0.01	0.11 ± 0.06	0.01 ± 0.01	0.08 ± 0.07	0.04 ± 0.00	0.03 ± 0.02	0.11 ± 0.01	0.01 ± 0.01	0.02 ± 0.01	0.03 ± 0.02	0.10 ± 0.03	0.01 ± 0.00
V20	0.19 ± 0.09 ^b^	0.04 ± 0.02 ^b^	0.03 ± 0.03 ^b^	0.12 ± 0.05 ^b^	0.06 ± 0.01 ^b^	0.10 ± 0.02 ^b^	0.09 ± 0.05 ^b^	0.25 ± 0.31 ^b^	0.15 ± 0.21 ^b^	0.04 ± 0.00 ^b^	0.16 ± 0.00 ^b^	0.35 ± 0.04 ^b^	0.45 ± 0.20 ^ab^	0.14 ± 0.04 ^b^	0.87 ± 0.03 ^a^
V21	0.18 ± 0.18	0.02 ± 0.01	0.04 ± 0.03	0.07 ± 0.03	0.06 ± 0.06	0.03 ± 0.01	0.05 ± 0.01	0.03 ± 0.02	0.16 ± 0.12	0.04 ± 0.01	0.06 ± 0.00	0.05 ± 0.00	0.07 ± 0.05	0.05 ± 0.00	0.00 ± 0.00
V22	0.28 ± 0.19	0.01 ± 0.00	0.34 ± 0.02	0.18 ± 0.06	0.21 ± 0.08	0.30 ± 0.09	0.17 ± 0.04	0.22 ± 0.17	0.10 ± 0.02	0.15 ± 0.01	0.16 ± 0.01	0.12 ± 0.03	0.31 ± 0.18	0.15 ± 0.07	0.02 ± 0.01
V23	0.94 ± 0.12 ^a^	0.21 ± 0.10 ^d^	0.17 ± 0.07 ^d^	0.50 ± 0.20 ^abcd^	0.40 ± 0.07 ^bcd^	0.33 ± 0.33 ^cd^	0.40 ± 0.02 ^bcd^	0.42 ± 0.07 ^bcd^	0.45 ± 0.02 ^abcd^	0.83 ± 0.14 ^ab^	0.35 ± 0.08 ^bcd^	0.72 ± 0.06 ^abc^	0.47 ± 0.04 ^abcd^	0.63 ± 0.11 ^abcd^	0.30 ± 0.01 ^cd^
V24	0.18 ± 0.14 ^bcde^	0.02 ± 0.01 ^e^	0.06 ± 0.03 ^de^	0.12 ± 0.05 ^cde^	0.03 ± 0.02 ^e^	0.05 ± 0.00 ^de^	0.06 ± 0.04 ^de^	0.10 ± 0.08 ^cde^	0.34 ± 0.01 ^abcd^	0.02 ± 0.00 ^e^	0.14 ± 0.01 ^cde^	0.48 ± 0.02 ^ab^	0.50 ± 0.02 ^a^	0.08 ± 0.03 ^cde^	0.37 ± 0.02 ^abc^
V25	0.01 ± 0.00 ^c^	0.00 ± 0.00 ^c^	0.01 ± 0.01 ^bc^	0.04 ± 0.01 ^abc^	0.05 ± 0.03 ^a^	0.01 ± 0.00 ^bc^	0.05 ± 0.00 ^ab^	0.01 ± 0.01 ^bc^	0.02 ± 0.00 ^abc^	0.01 ± 0.01 ^bc^	0.05 ± 0.01 ^ab^	0.02 ± 0.01 ^abc^	0.01 ± 0.00 ^bc^	0.02 ± 0.01 ^bc^	0.01 ± 0.00 ^bc^
V26	0.02 ± 0.01 ^bc^	0.01 ± 0.01 ^c^	0.01 ± 0.01 ^bc^	0.04 ± 0.02 ^bc^	0.04 ± 0.03 ^bc^	0.01 ± 0.00 ^bc^	0.12 ± 0.02 ^a^	0.01 ± 0.01 ^bc^	0.03 ± 0.01 ^bc^	0.02 ± 0.01 ^bc^	0.03 ± 0.00 ^bc^	0.04 ± 0.02 ^bc^	0.06 ± 0.01 ^b^	0.05 ± 0.00 ^bc^	0.01 ± 0.01 ^c^
V27	0.35 ± 0.08 ^ab^	0.07 ± 0.05 ^b^	0.28 ± 0.01 ^ab^	0.07 ± 0.05 ^b^	0.42 ± 0.13	0.25 ± 0.06 ^ab^	0.36 ± 0.05 ^ab^	0.61 ± 0.33 ^a^	0.07 ± 0.00 ^b^	0.29 ± 0.01 ^ab^	0.06 ± 0.01 ^b^	0.09 ± 0.01 ^b^	0.07 ± 0.10 ^ab^	0.26 ± 0.03 ^ab^	0.57 ± 0.05 ^a^
V28	0.03 ± 0.03 ^ab^	0.00 ± 0.00 ^b^	0.01 ± 0.01 ^ab^	0.01 ± 0.01 ^ab^	0.06 ± 0.01 ^ab^	0.01 ± 0.01 ^ab^	0.04 ± 0.03 ^ab^	0.08 ± 0.01 ^a^	0.00 ± 0.00 ^ab^	0.01 ± 0.01 ^ab^	0.02 ± 0.02 ^ab^	0.00 ± 0.00 ^b^	0.04 ± 0.05 ^ab^	0.04 ± 0.02 ^ab^	0.00 ± 0.01 ^ab^
V29	0.06 ± 0.02 ^ab^	0.01 ± 0.00 ^b^	0.04 ± 0.03 ^ab^	0.03 ± 0.03 ^ab^	0.06 ± 0.01 ^ab^	0.03 ± 0.00 ^ab^	0.07 ± 0.04 ^ab^	0.14 ± 0.14 ^ab^	0.04 ± 0.02 ^ab^	0.08 ± 0.01 ^ab^	0.08 ± 0.01 ^ab^	0.19 ± 0.04 ^a^	0.06 ± 0.00 ^ab^	0.07 ± 0.06 ^ab^	0.08 ± 0.02 ^ab^
V30	0.07 ± 0.03 ^bcd^	0.02 ± 0.01 ^d^	0.02 ± 0.01 ^d^	0.03 ± 0.03 ^d^	0.06 ± 0.00 ^bcd^	0.05 ± 0.01 ^bcd^	0.07 ± 0.04 ^bcd^	0.18 ± 0.22 ^abcd^	0.24 ± 0.02 ^abcd^	0.03 ± 0.00 ^cd^	0.11 ± 0.01 ^bcd^	0.38 ± 0.02 ^a^	0.29 ± 0.06 ^ab^	0.10 ± 0.07 ^bcd^	0.29 ± 0.03 ^abc^
V31	0.01 ± 0.01 ^bc^	0.01 ± 0.00 ^c^	0.05 ± 0.02 ^bc^	0.01 ± 0.00 ^bc^	0.11 ± 0.08 ^ab^	0.02 ± 0.01 ^bc^	0.08 ± 0.03 ^abc^	0.05 ± 0.02 ^bc^	0.04 ± 0.01 ^bc^	0.01 ± 0.00 ^bc^	0.02 ± 0.02 ^bc^	0.10 ± 0.01 ^abc^	0.17 ± 0.01 ^a^	0.10 ± 0.03 ^abc^	0.03 ± 0.01 ^bc^
V32	0.81 ± 0.01 ^ab^	0.05 ± 0.05 ^b^	0.44 ± 0.04 ^ab^	0.15 ± 0.07 ^b^	0.35 ± 0.08 ^b^	0.32 ± 0.20 ^b^	0.37 ± 0.03 ^b^	1.22 ± 0.73 ^a^	0.01 ± 0.001 ^b^	0.44 ± 0.01 ^ab^	0.09 ± 0.05 ^b^	0.11 ± 0.02 ^b^	0.11 ± 0.05 ^b^	0.62 ± 0.28 ^ab^	0.12 ± 0.02 ^b^
V33	0.01 ± 0.00	0.01 ± 0.01	0.04 ± 0.05	0.04 ± 0.00	0.12 ± 0.03	0.03 ± 0.03	0.20 ± 0.00	0.14 ± 0.16	0.00 ± 0.00	0.01 ± 0.00	0.01 ± 0.01	0.06 ± 0.07	0.20 ± 0.27	0.07 ± 0.05	0.00 ± 0.00
V34	0.64 ± 0.39	0.07 ± 0.05	0.16 ± 0.03	0.35 ± 0.41	0.18 ± 0.03	0.21 ± 0.10	0.20 ± 0.04	0.55 ± 0.68	0.76 ± 0.07	0.19 ± 0.04	0.31 ± 0.06	1.00 ± 0.07	0.95 ± 0.18	0.54 ± 0.03	0.88 ± 0.09
V35	0.13 ± 0.07	0.09 ± 0.00	0.10 ± 0.06	0.96 ± 0.72	0.18 ± 0.09	0.11 ± 0.00	0.90 ± 1.00	0.31 ± 0.19	1.22 ± 0.30	0.06 ± 0.04	0.45 ± 0.15	0.28 ± 0.04	0.81 ± 0.08	0.15 ± 0.02	0.76 ± 0.04
V36	0.18 ± 0.02 ^cd^	0.12 ± 0.03 ^def^	0.03 ± 0.00 ^f^	0.17 ± 0.02 ^cd^	0.07 ± 0.01 ^def^	0.28 ± 0.02 ^bc^	0.04 ± 0.00 ^ef^	0.15 ± 0.02 ^de^	0.12 ± 0.05 ^def^	0.08 ± 0.00 ^def^	0.10 ± 0.01 ^def^	0.11 ± 0.00 ^def^	0.31 ± 0.08	0.04 ± 0.01 ^ef^	0.44 ± 0.04 ^a^
V37	0.01 ± 0.00 ^e^	0.05 ± 0.00 ^bc^	0.08 ± 0.01 ^a^	0.03 ± 0.01 ^cde^	0.04 ± 0.00 ^bcd^	0.04 ± 0.01 ^bcde^	0.04 ± 0.00 ^bcde^	0.03 ± 0.01 ^cde^	0.02 ± 0.00 ^e^	0.02 ± 0.00 ^de^	0.02 ± 0.00 ^de^	0.02 ± 0.00 ^e^	0.05 ± 0.01 ^ab^	0.04 ± 0.00 ^bcd^	0.03 ± 0.00 ^bcde^
V38	0.36 ± 0.06 ^bcd^	0.47 ± 0.03 ^ab^	0.57 ± 0.05 ^a^	0.18 ± 0.01 ^fg^	0.46 ± 0.03 ^ab^	0.27 ± 0.05 ^cdef^	0.42 ± 0.00 ^abc^	0.24 ± 0.06 ^defg^	0.15 ± 0.05 ^fg^	0.20 ± 0.04 ^efg^	0.12 ± 0.01 ^fg^	0.10 ± 0.01 ^g^	0.25 ± 0.01 ^defg^	0.39 ± 0.06 ^bcd^	0.35 ± 0.04 ^bcde^
V39	0.87 ± 0.02 ^efg^	1.50 ± 0.0 ^ab^	1.71 ± 0.14 ^a^	0.73 ± 0.03 ^fgh^	1.43 ± 0.02 ^ab^	0.94 ± 0.09 ^cdef^	1.36 ± 0.01 ^abc^	0.86 ± 0.16 ^efg^	0.51 ± 0.13 ^gh^	0.63 ± 0.12 ^fgh^	0.69 ± 0.12 ^fgh^	0.36 ± 0.07	0.90 ± 0.09 ^defg^	1.21 ± 0.13 ^bcde^	1.28 ± 0.14 ^bcd^
V40	12.08 ± 0.22 ^efg^	25.54 ± 2.35 ^a^	20.84 ± 2.10 ^abc^	12.71 ± 1.78 ^efg^	23.14 ± 1.34 ^ab^	18.17 ± 0.38 ^bcd^	19.57 ± 0.05 ^bcd^	16.66 ± 0.24 ^cde^	10.44 ± 1.54 ^fg^	12.20 ± 1.78 ^efg^	7.44 ± 1.24 ^g^	7.64 ± 0.13 ^g^	14.20 ± 1.58 ^def^	18.94 ± 1.40 ^bcd^	9.75 ± 0.98 ^fg^
V41	0.79 ± 0.10 ^bcd^	1.21 ± 0.02 ^b^	0.72 ± 0.05 ^bcd^	0.75 ± 0.15 ^bcd^	1.02 ± 0.02 ^bc^	1.00 ± 0.13 ^bc^	0.80 ± 0.10 ^bcd^	1.00 ± 0.23 ^bc^	0.63 ± 0.20 ^cd^	0.54 ± 0.07 ^cd^	0.44 ± 0.07 ^d^	0.49 ± 0.11 ^g^	0.87 ± 0.08 ^bcd^	0.77 ± 0.13 ^bcd^	1.91 ± 0.29 ^a^
V42	21.72 ± 0.66 ^defg^	46.10 ± 0.16 ^a^	33.84 ± 2.81 ^bc^	20.85 ± 5.05 ^defg^	45.79 ± 2.37 ^a^	25.63 ± 3.83 ^cdef^	28.79 ± 2.07 ^bcde^	38.53 ± 1.28 ^ab^	17.81 ± 3.80 ^efg^	22.35 ± 3.13 ^defg^	12.01 ± 3.45 ^g^	11.58 ± 1.07 ^g^	15.72 ± 1.17 ^fg^	29.11 ± 4.09 ^bcd^	17.21 ± 1.71 ^fg^
V43	0.35 ± 0.13 ^ab^	0.10 ± 0.13 ^abc^	0.16 ± 0.08 ^abc^	0.02 ± 0.00 ^c^	0.17 ± 0.02 ^abc^	0.24 ± 0.07 ^abc^	0.21 ± 0.04 ^abc^	0.11 ± 0.12 ^abc^	0.17 ± 0.04 ^abc^	0.08 ± 0.11 ^bc^	0.01 ± 0.01 ^c^	0.09 ± 0.00 ^abc^	0.26 ± 0.00 ^abc^	0.23 ± 0.05 ^abc^	0.38 ± 0.08 ^a^
V44	1.55 ± 0.19 ^bcd^	1.60 ± 0.33 ^bcd^	2.92 ± 0.56 ^a^	1.11 ± 0.26 ^bcd^	1.32 ± 0.14 ^bcd^	1.69 ± 0.66 ^bc^	1.13 ± 0.23 ^bcd^	1.34 ± 0.18 ^bcd^	0.77 ± 0.10 ^cd^	0.75 ± 0.01 ^cd^	0.82 ± 0.13 ^cd^	0.47 ± 0.06 ^d^	0.99 ± 0.14 ^bcd^	1.39 ± 0.11 ^bcd^	2.03 ± 0.38 ^ab^
V45	0.06 ± 0.03 ^bc^	0.21 ± 0.02 ^ab^	0.25 ± 0.07 ^a^	0.09 ± 0.00 ^abc^	0.13 ± 0.04 ^abc^	0.07 ± 0.09 ^bc^	0.16 ± 0.02 ^abc^	0.12 ± 0.08 ^abc^	0.08 ± 0.02 ^bc^	0.10 ± 0.02 ^abc^	0.07 ± 0.00 ^bc^	0.04 ± 0.00 ^c^	0.15 ± 0.01 ^abc^	0.18 ± 0.03 ^abc^	0.22 ± 0.04 ^ab^
V46	3.18 ± 0.51 ^b^	0.85 ± 0.26 ^c^	5.69 ± 0.58 ^a^	1.17 ± 0.50 ^c^	1.00 ± 0.18 ^c^	0.56 ± 0.18 ^c^	0.47 ± 0.24 ^c^	0.93 ± 0.32 ^c^	0.51 ± 0.03 ^c^	0.36 ± 0.02 ^c^	1.05 ± 0.30 ^c^	0.78 ± 0.09 ^c^	0.76 ± 0.03 ^c^	1.29 ± 0.31 ^c^	1.09 ± 0.20 ^c^
V47	0.00 ± 0.00 ^c^	0.31 ± 0.05 ^ab^	0.16 ± 0.18 ^abc^	0.21 ± 0.06 ^abc^	0.31 ± 0.03 ^ab^	0.38 ± 0.19 ^ab^	0.34 ± 0.01 ^ab^	0.35 ± 0.02 ^ab^	0.14 ± 0.01 ^bc^	0.17 ± 0.04 ^abc^	0.11 ± 0.08 ^bc^	0.10 ± 0.00 ^bc^	0.27 ± 0.04 ^abc^	0.25 ± 0.05 ^abc^	0.46 ± 0.33 ^a^
V48	5.19 ± 1.18 ^b^	1.57 ± 0.36 ^c^	8.41 ± 1.02 ^a^	1.69 ± 0.68 ^c^	1.53 ± 0.18 ^c^	1.05 ± 0.02 ^c^	1.31 ± 0.39 ^c^	1.76 ± 0.66 ^c^	1.06 ± 0.03 ^c^	0.74 ± 0.05 ^c^	1.55 ± 0.41 ^c^	0.98 ± 0.21 ^c^	1.38 ± 0.00 ^c^	2.56 ± 0.55 ^c^	2.28 ± 0.37 ^c^
V49	0.61 ± 0.02 ^bc^	1.07 ± 0.28 ^abc^	0.96 ± 0.16 ^abc^	0.72 ± 0.26 ^abc^	1.04 ± 0.13 ^abc^	1.20 ± 0.63 ^abc^	0.85 ± 0.23 ^abc^	1.28 ± 0.25 ^ab^	0.59 ± 0.06 ^bc^	0.61 ± 0.05 ^bc^	0.46 ± 0.15 ^bc^	0.31 ± 0.06 ^c^	0.78 ± 0.04 ^abc^	0.91 ± 0.16 ^abc^	1.55 ± 0.26 ^a^
V50	1.81 ± 0.25 ^ab^	2.09 ± 0.60 ^ab^	2.24 ± 0.32 ^ab^	1.36 ± 0.49 ^ab^	2.15 ± 0.50 ^ab^	2.39 ± 1.41 ^ab^	1.62 ± 0.41 ^ab^	2.13 ± 0.41 ^ab^	1.10 ± 0.07 ^ab^	1.06 ± 0.15 ^ab^	0.89 ±0.36 ^b^	0.56 ± 0.07 ^b^	1.50 ± 0.02 ^ab^	1.81 ± 0.30 ^ab^	3.03 ± 0.39 ^a^
V51	0.04 ± 0.00 ^c^	0.26 ± 0.08 ^abc^	0.38 ± 0.03 ^a^	0.16 ± 0.00 ^abc^	0.18 ± 0.16 ^abc^	0.09 ± 0.09 ^bc^	0.31 ± 0.02 ^ab^	0.17 ± 0.01 ^abc^	0.14 ± 0.00 ^abc^	0.16 ± 0.03 ^abc^	0.10 ± 0.00 ^bc^	0.05 ± 0.02 ^c^	0.05 ± 0.07 ^c^	0.31 ± 0.01 ^ab^	0.11 ± 0.11 ^bc^
V52	0.07 ± 0.01 ^b^	0.14 ± 0.04 ^ab^	0.16 ± 0.03 ^ab^	0.09 ± 0.04 ^ab^	0.13 ± 0.02 ^ab^	0.17 ± 0.09 ^ab^	0.13 ± 0.04 ^ab^	0.13 ± 0.03 ^ab^	0.09 ± 0.00 ^ab^	0.06 ± 0.00 ^b^	0.05 ± 0.03 ^b^	0.04 ± 0.00 ^b^	0.10 ± 0.01 ^ab^	0.13 ± 0.02 ^ab^	0.19 ± 0.01 ^a^
V53	1.45 ± 0.38 ^a^	0.20 ± 0.10 ^c^	0.19 ± 0.03 ^c^	0.12 ± 0.06 ^c^	0.17 ± 0.03 ^c^	0.20 ± 0.13 ^c^	0.14 ± 0.04 ^c^	0.24 ± 0.09 ^c^	0.11 ± 0.01 ^c^	0.09 ± 0.01 ^c^	0.08 ± 0.03 ^c^	0.06 ± 0.01 ^c^	0.15 ± 0.01 ^c^	0.16 ± 0.02 ^c^	0.90 ± 0.13 ^b^
V54	1.30 ± 0.37 ^a^	0.08 ± 0.05 ^c^	0.09 ± 0.02 ^c^	0.03 ± 0.02 ^c^	0.06 ± 0.02 ^c^	0.06 ± 0.04 ^c^	0.05 ± 0.02 ^c^	0.09 ± 0.05 ^c^	0.03 ± 0.00 ^c^	0.03 ± 0.00 ^c^	0.03 ± 0.01 ^c^	0.01 ± 0.00 ^c^	0.01 ± 0.01 ^c^	0.07 ± 0.01 ^c^	0.66 ± 0.12 ^b^
V55	0.10 ± 0.03	0.09 ± 0.07	0.03 ± 0.00	0.05 ± 0.02	0.49 ± 0.61	0.06 ± 0.06	0.05 ± 0.03	0.10 ± 0.12	0.04 ± 0.02	0.04 ± 0.02	0.06 ± 0.04	0.01 ± 0.00	0.04 ± 0.01	0.07 ± 0.03	0.03 ± 0.00
V56	0.03 ± 0.00	0.05 ± 0.03	0.01 ± 0.00	0.02 ± 0.01	0.05 ± 0.00	0.02 ± 0.02	0.02 ± 0.02	0.55 ± 0.76	0.02 ± 0.01	0.02 ± 0.00	0.03 ± 0.02	0.00 ± 0.00	0.01 ± 0.00	0.04 ± 0.02	0.01 ± 0.00
V57	0.20 ± 0.02	0.19 ± 0.18	0.04 ± 0.01	0.14 ± 0.10	0.24 ± 0.00	0.16 ± 0.16	0.12 ± 0.08	0.31 ± 0.42	0.11 ± 0.06	0.15 ± 0.01	0.15 ± 0.10	0.02 ± 0.01	0.04 ± 0.01	0.20 ± 0.08	0.04 ± 0.00

Values in a row with different letters are significantly different (Tukey test, *p* < 0.05).

## Supplementary Materials

The following are available online at https://www.mdpi.com/2304-8158/9/5/541/s1, Figure S1: Headspace SPME-GC-MS profile (TIC, total ion current) of the of the Northern Brewer cultivar, Figure S2: Comparison among the headspace SPME-GC-MS profiles (TIC, total ion current) of fifteen hop varieties cultivated in the Marche region, Italy.
