# Supplementary material for: Brewing Quality of Hop Varieties Cultivated in Central Italy Based on Multivolatile Fingerprinting and Bitter Acid Content
Source: Foods. 2020 Apr 29;9(5):541. doi: 10.3390/foods9050541 (PMC7278677; doi:10.3390/foods9050541)
Supplement: Supplementary file 1 [file foods-09-00541-s001.pdf]

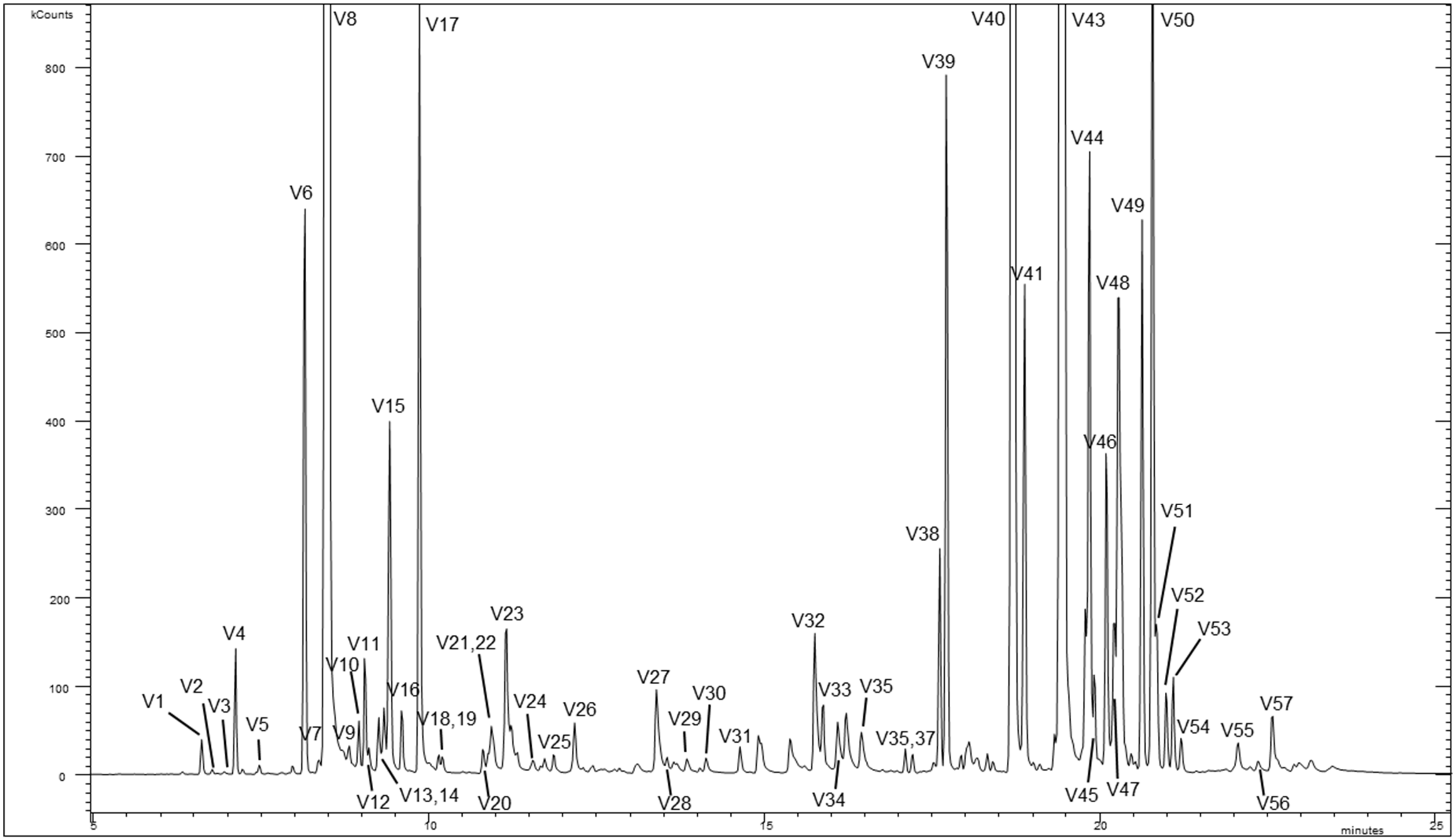

Figure S1. Headspace SPME-GC-MS profile (TIC, total ion current) of the of the Northern Brewer cultivar. Peak identification as in Table 3.

Figure S2. Comparison among the headspace SPME-GC-MS profiles (TIC, total ion current) of fifteen hop varieties cultivated in the Marche region, Italy. Peak V8 (β-myrcene) was chosen as reference (100% of full scale). Peak identification as in Table 3. (**a**) Hallertau; (**b**) Willamette; (**c**) Yeoman; (**d**) Centennial; (**e**) Fuggle; (**f**) Mount Hood; (**g**) Northern Brewer; (**h**) Galena; (**i**) Brewer's Gold; (**j**) Sterling; (**k**) Cascade; (**l**) Nugget; (**m**) Columbus; (**n**) Northdown; (**o**) Chinook.

S2 (a)

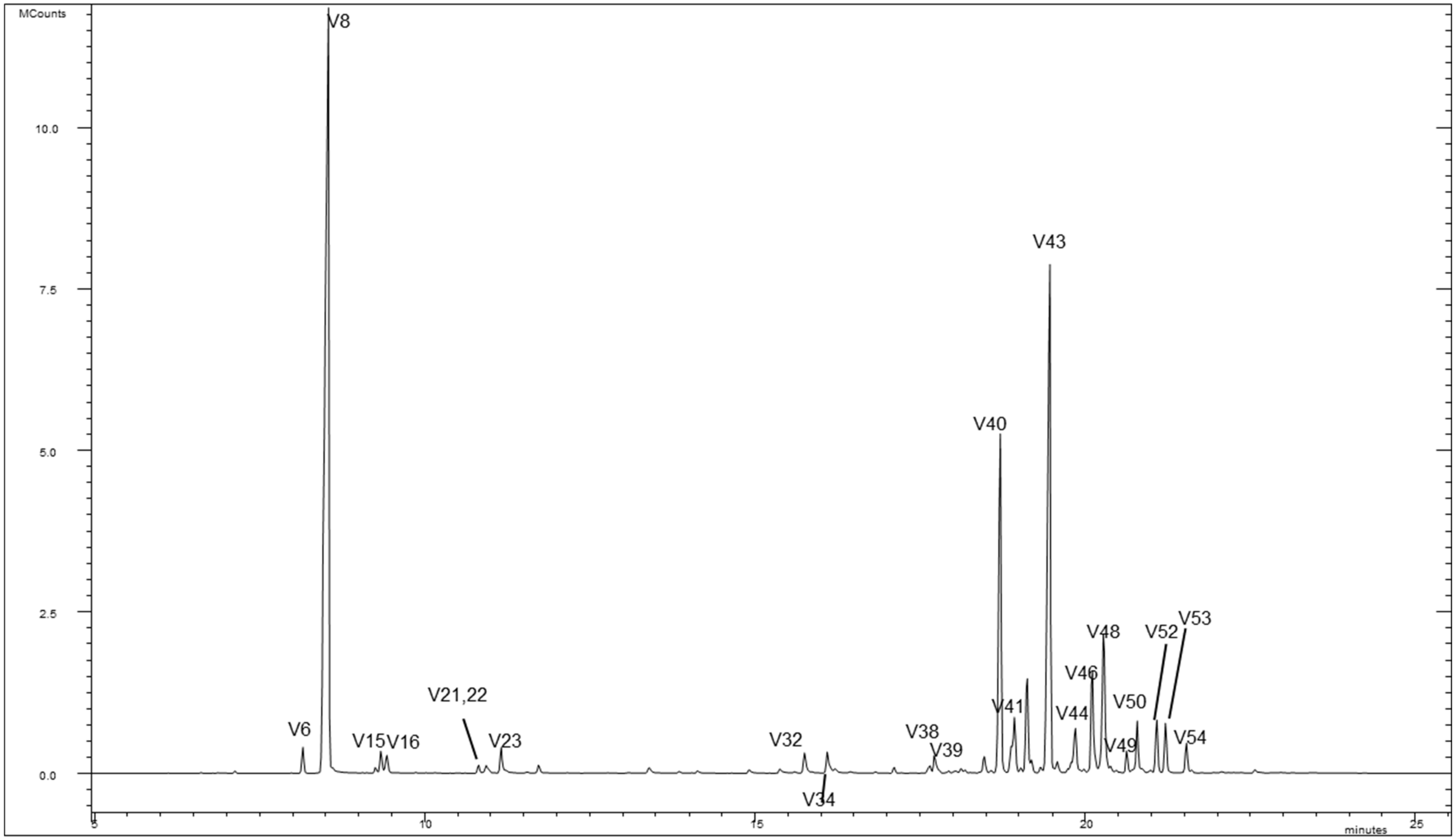

S2 (b)

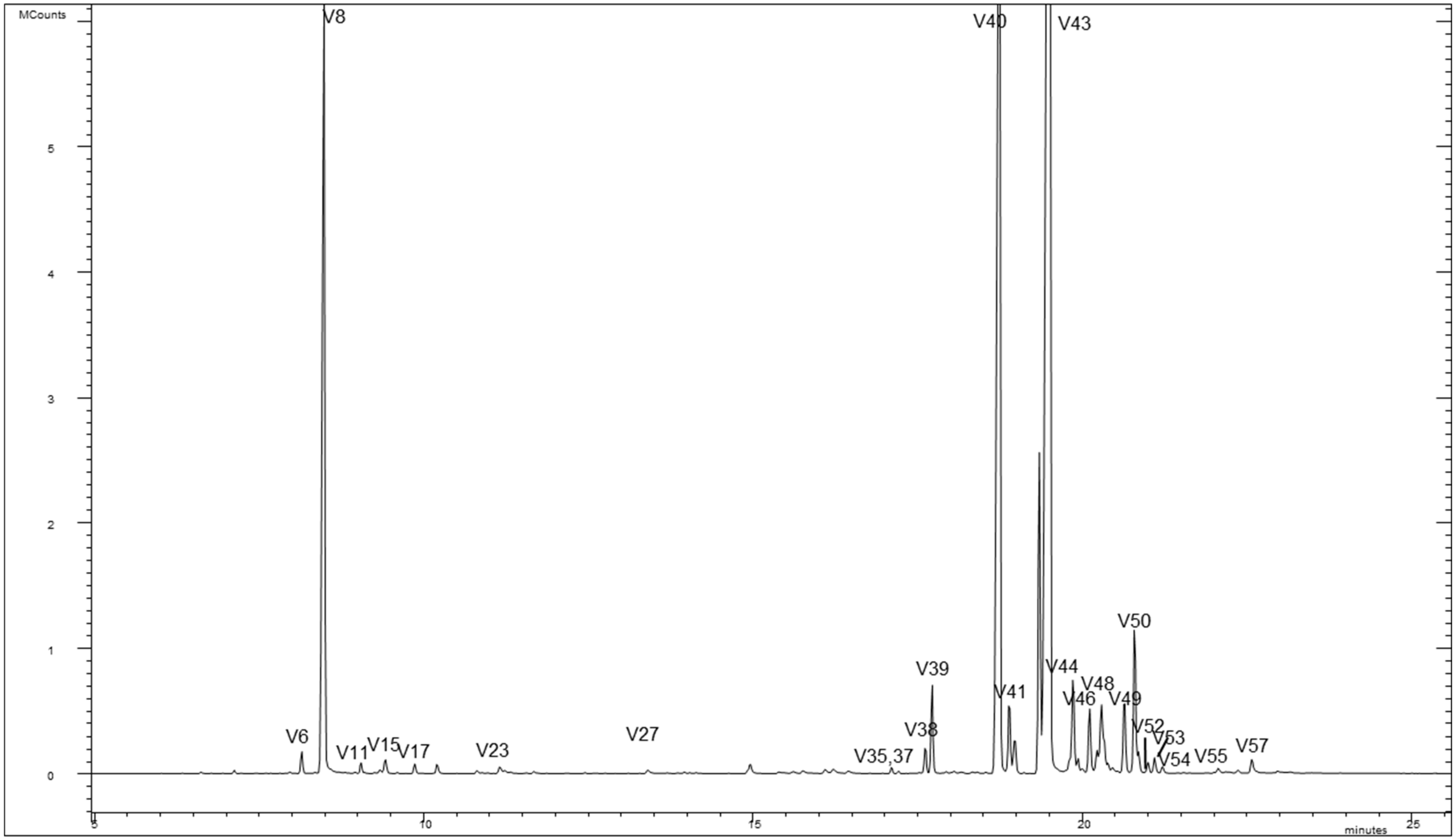

MCounts
5
4
3
2
1
0
V8
V40
V43
V50
V44
V48
V39
V41
V46
V49
V6
V11
V15
V17
V23
V27
V35,37
V38
V52
V53
V54
V55
V57
5
10
15
20
25
minutes

S2 (c)

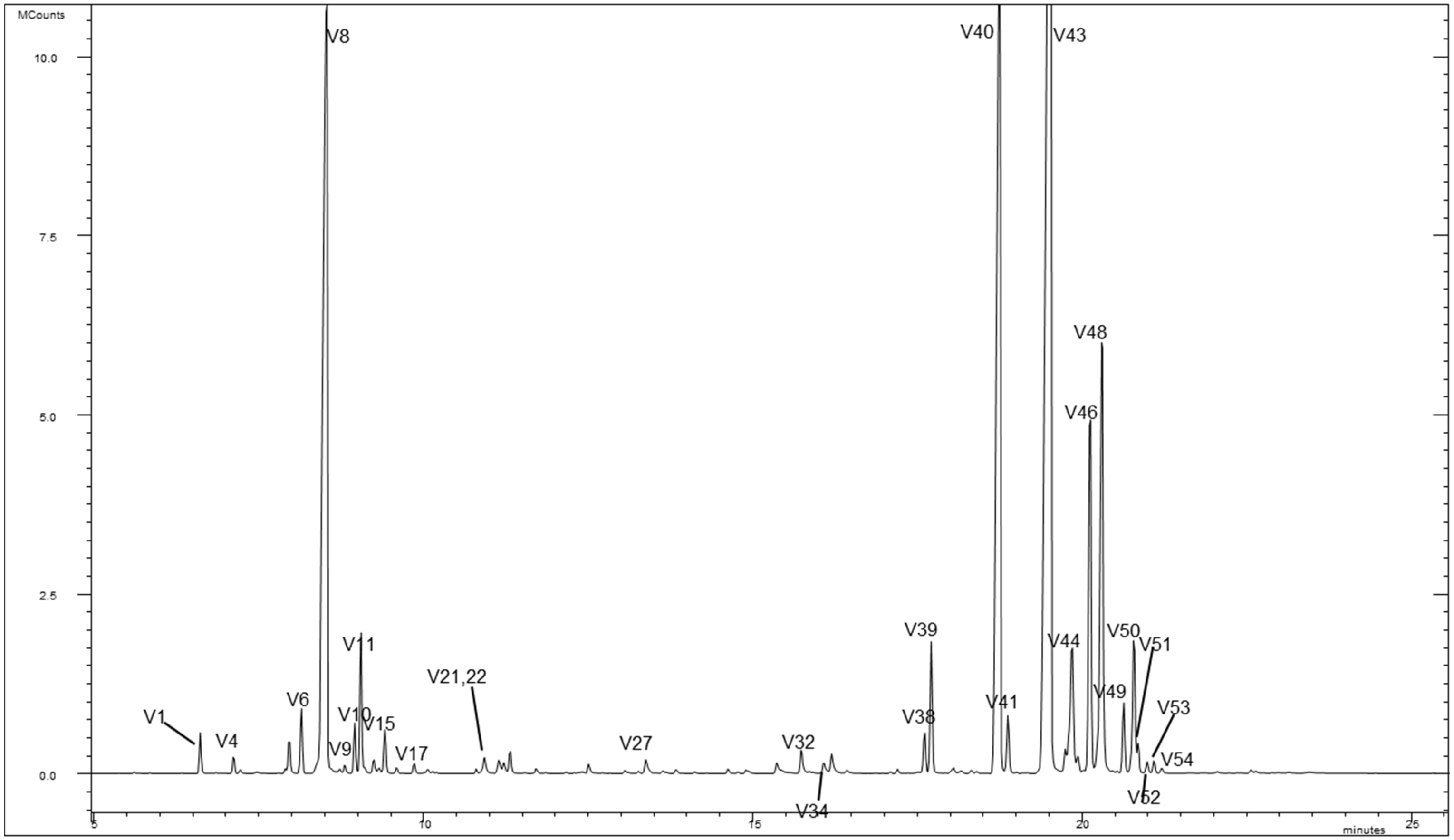

MCounts
10.0
7.5
5.0
2.5
0.0
5
10
15
20
25
minutes
V1
V4
V6
V8
V9
V10
V11
V15
V17
V21,22
V27
V32
V34
V38
V39
V40
V41
V43
V44
V46
V48
V49
V50
V51
V52
V53
V54

S2 (d)

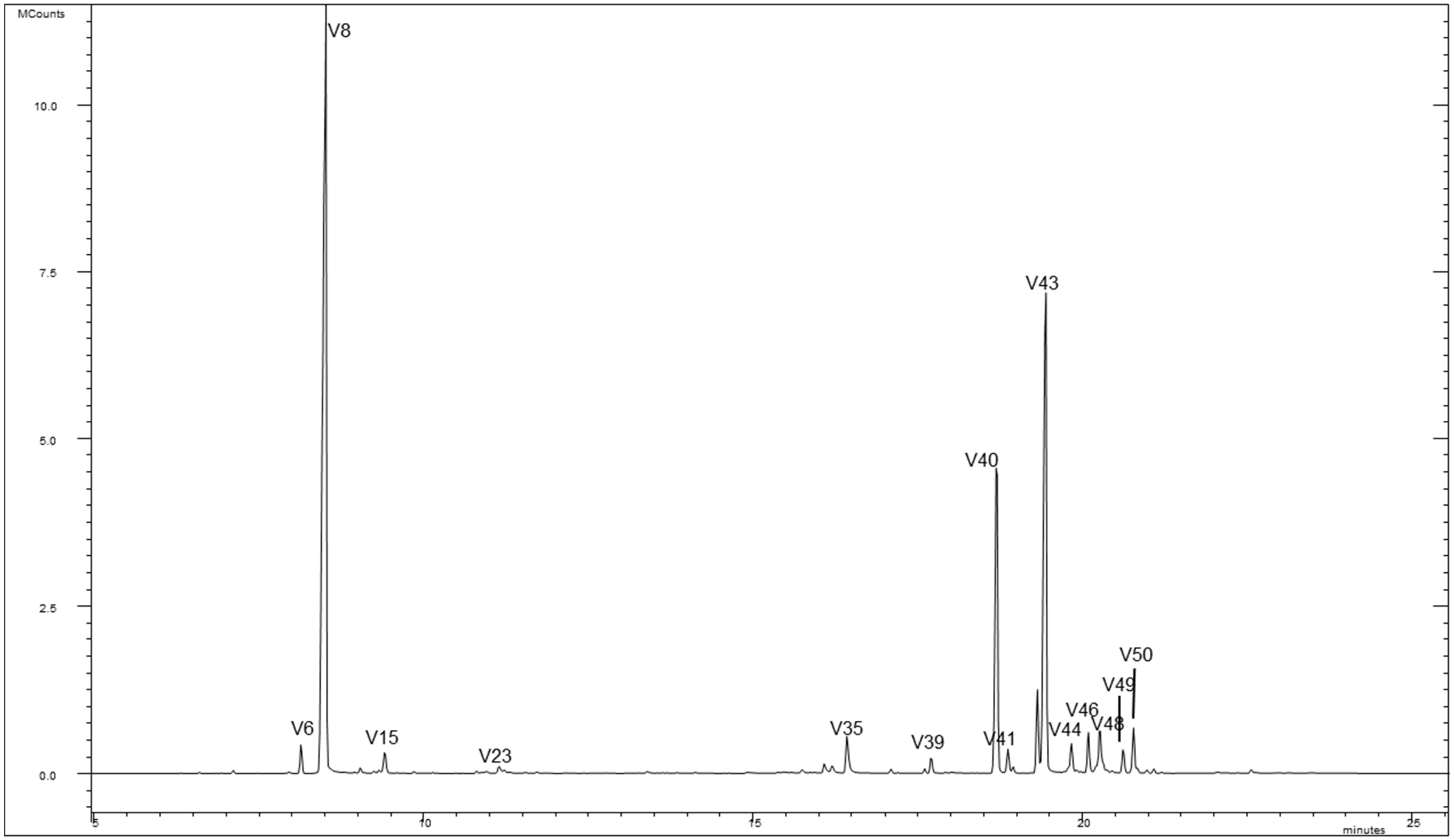
MCounts
10.0
7.5
5.0
2.5
0.0
V8
V6
V15
V23
V35
V39
V40
V41
V43
V44
V46
V48
V49
V50
5
10
15
20
25
minutes

S2 (e)

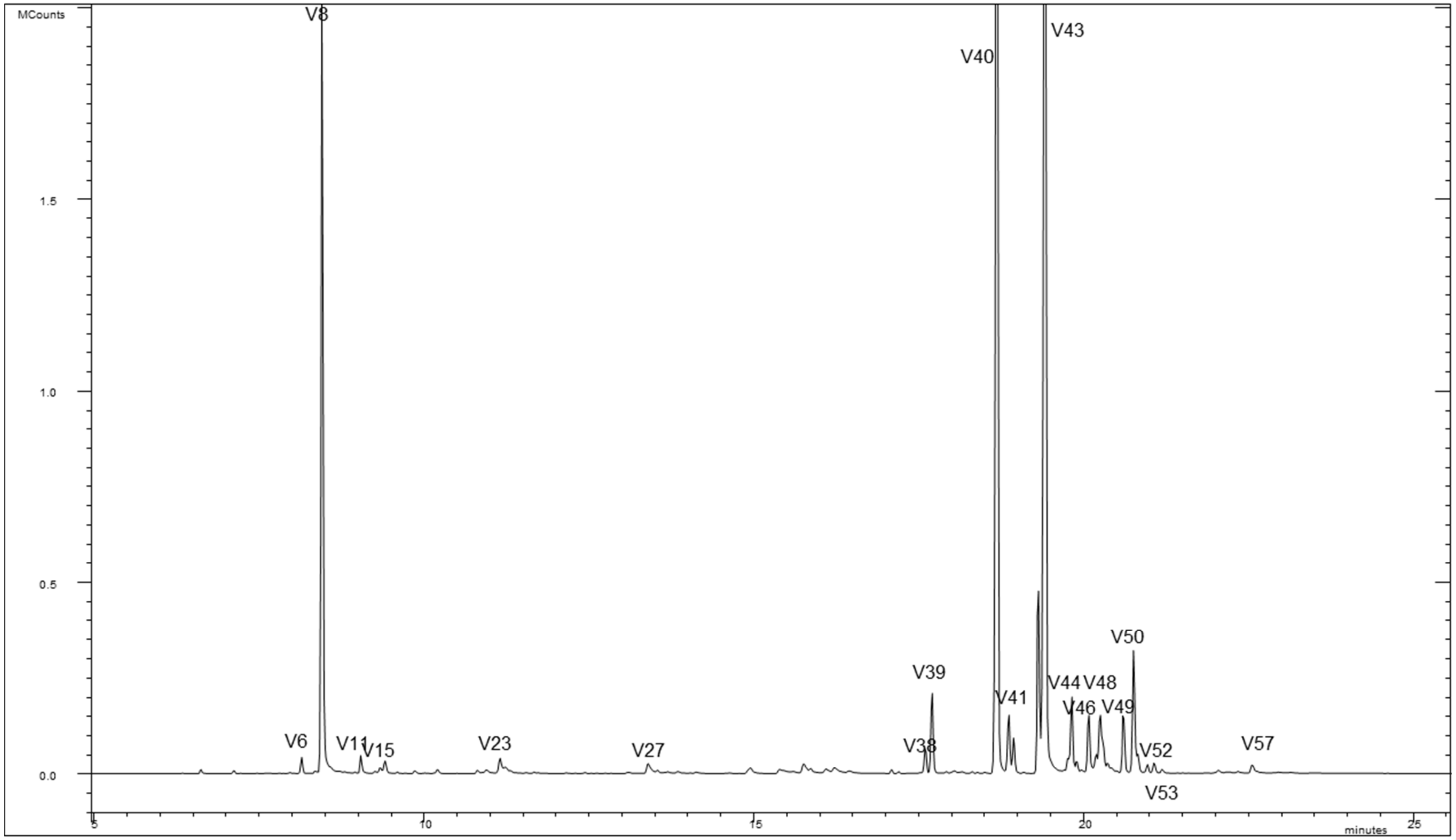

MCounts
V8
V40
V43
1.5
1.0
0.5
0.0
V39
V41
V44
V48
V50
V46
V49
V6
V11
V15
V23
V27
V38
V52
V57
V53
5
10
15
20
25
minutes

S2 (f)

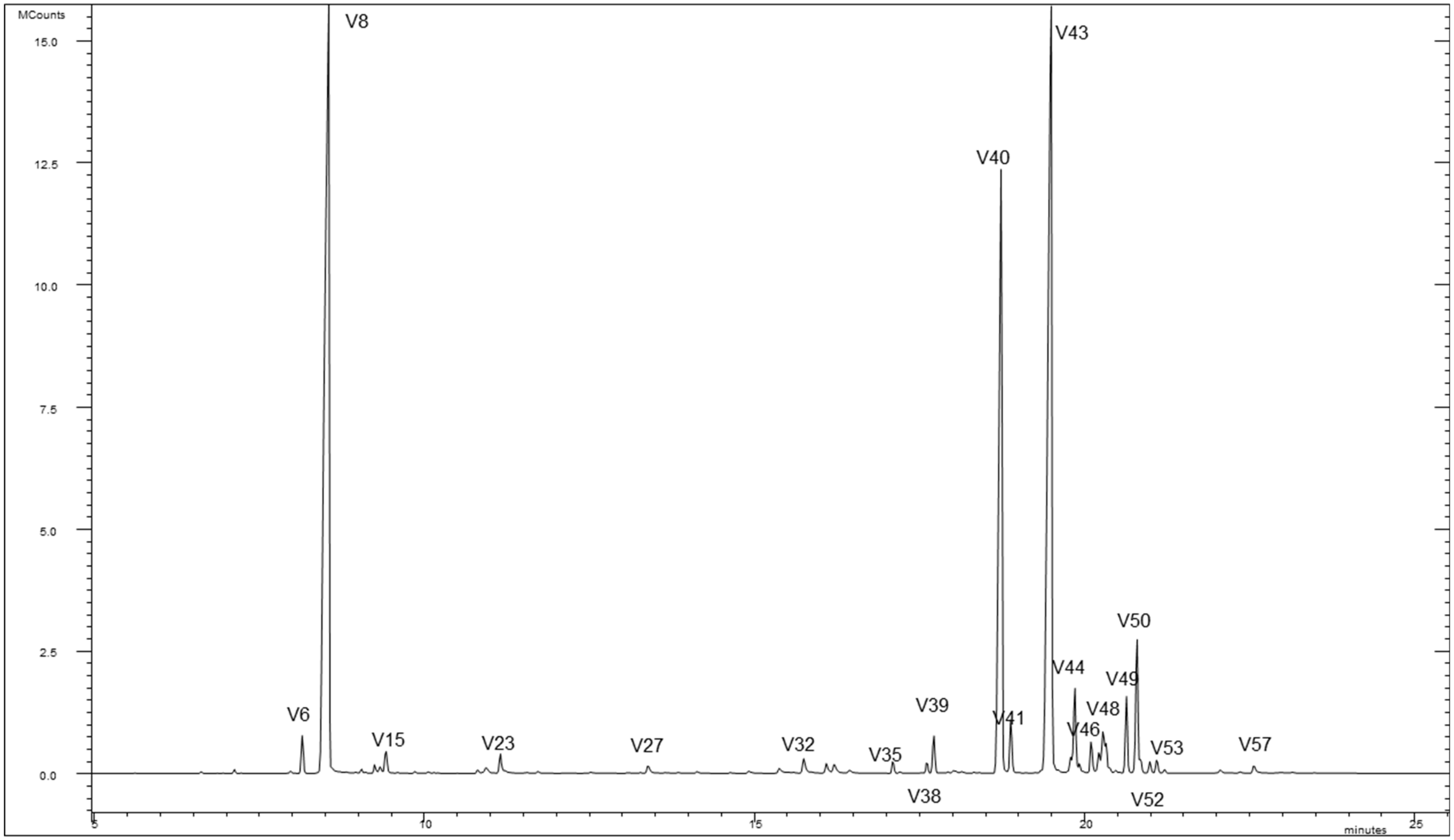

MCounts
15.0
12.5
10.0
7.5
5.0
2.5
0.0
V8
V43
V40
V50
V44
V49
V48
V39
V41
V46
V6
V15
V23
V27
V32
V35
V53
V57
V38
V52
5
10
15
20
25
minutes

S2 (g)

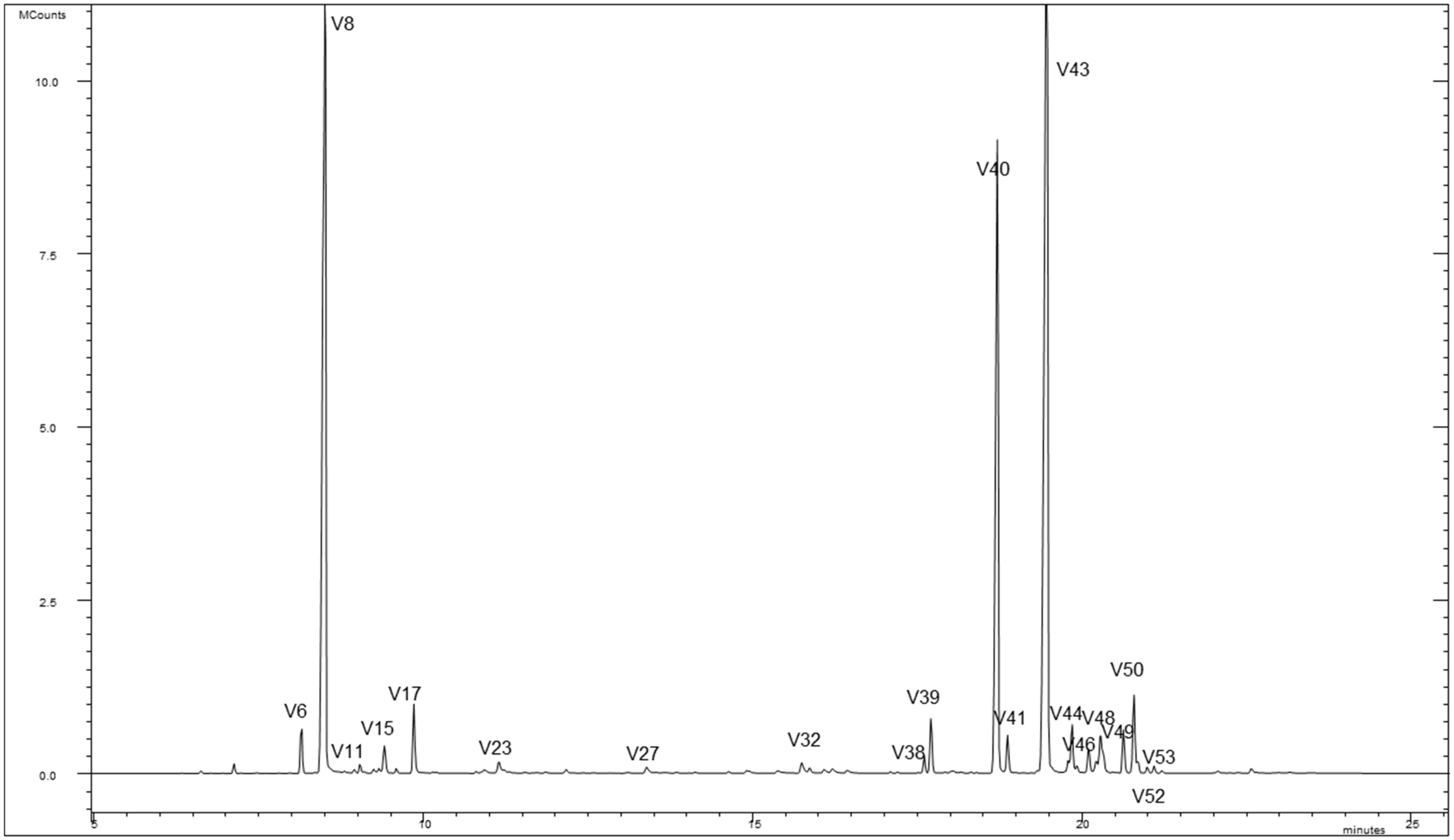

MCounts
10.0
7.5
5.0
2.5
0.0
V8
V43
V40
V50
V39
V6
V17
V44
V41
V48
V15
V49
V11
V23
V46
V32
V27
V38
V53
V52
5
10
15
20
25
minutes

S2 (h)

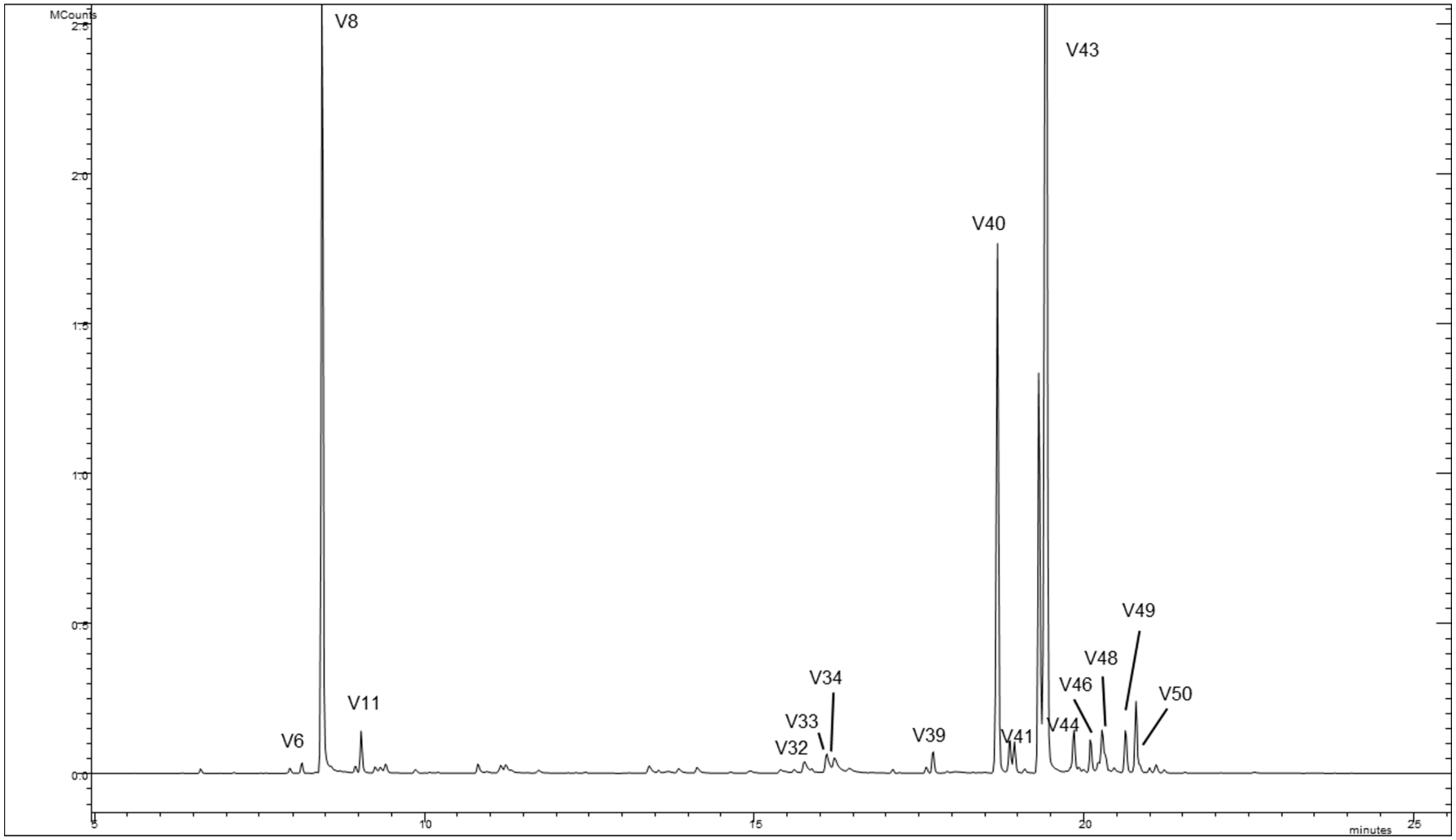
MCounts
2.5
2.0
1.5
1.0
0.5
0.0
5
10
15
20
25
minutes
V8
V43
V40
V49
V48
V46
V50
V34
V11
V33
V44
V6
V39
V41
V32

S2 (i)

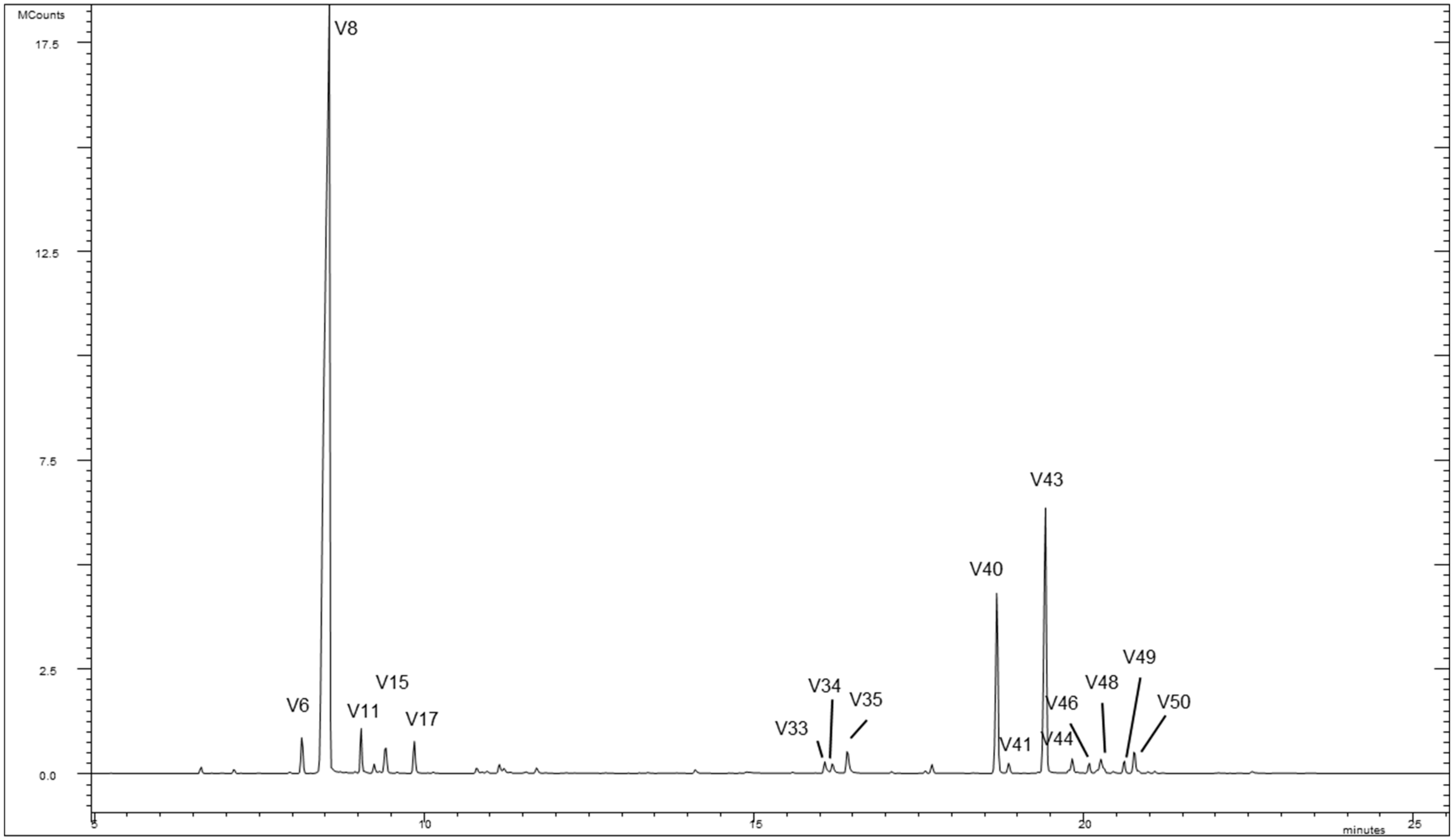

MCounts
17.5
12.5
7.5
2.5
0.0
5
10
15
20
25
minutes
V8
V6
V11
V15
V17
V33
V34
V35
V40
V41
V43
V44
V46
V48
V49
V50

S2 (j)

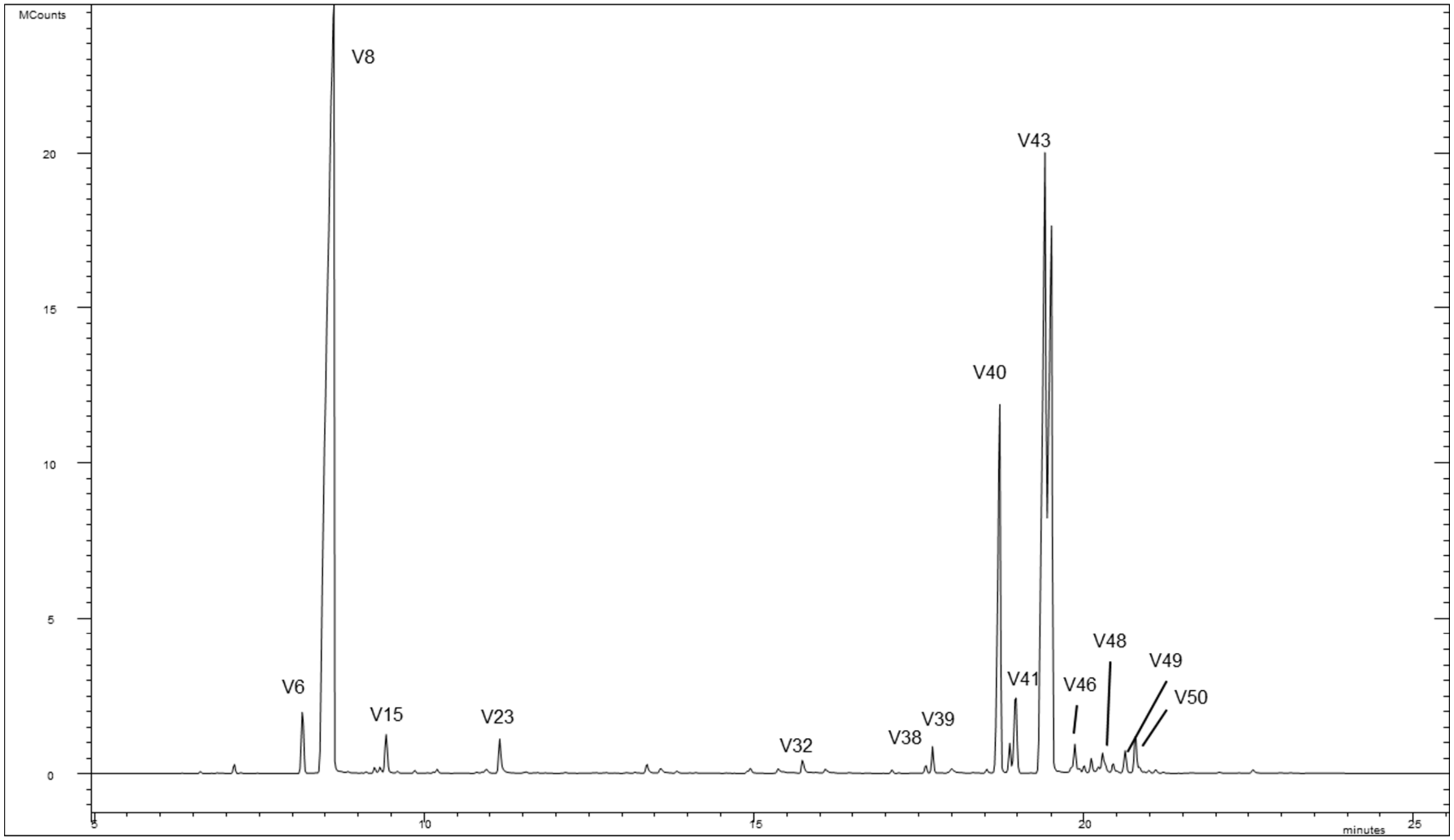
MCounts
V8
V43
V40
V6
V15
V23
V32
V38
V39
V41
V46
V48
V49
V50
20
15
10
5
0
5
10
15
20
minutes
25

S2 (k)

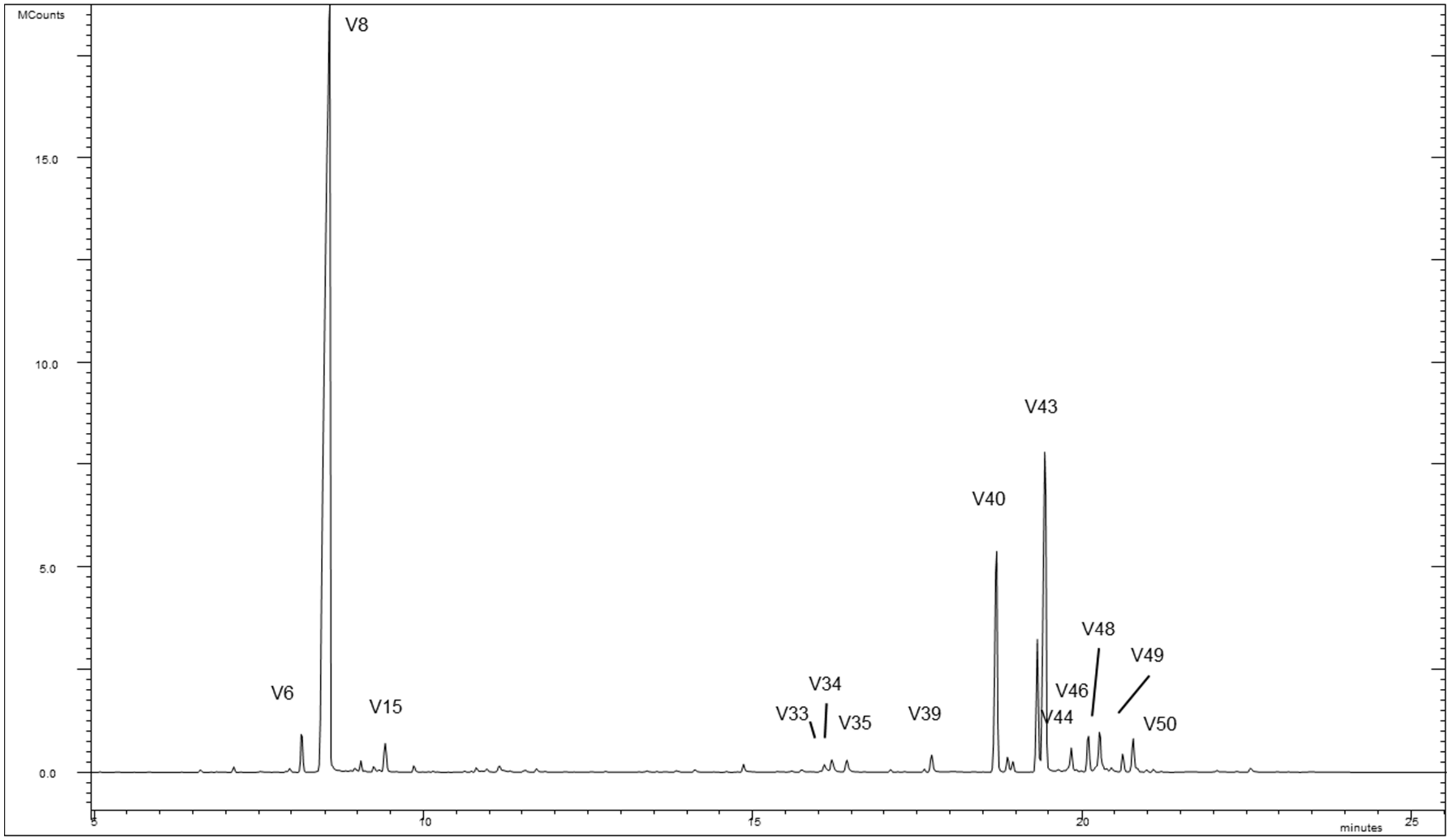
MCounts
15.0
10.0
5.0
0.0
V8
V6
V15
V33
V34
V35
V39
V40
V43
V44
V46
V48
V49
V50
5
10
15
20
25
minutes

S2 (l)

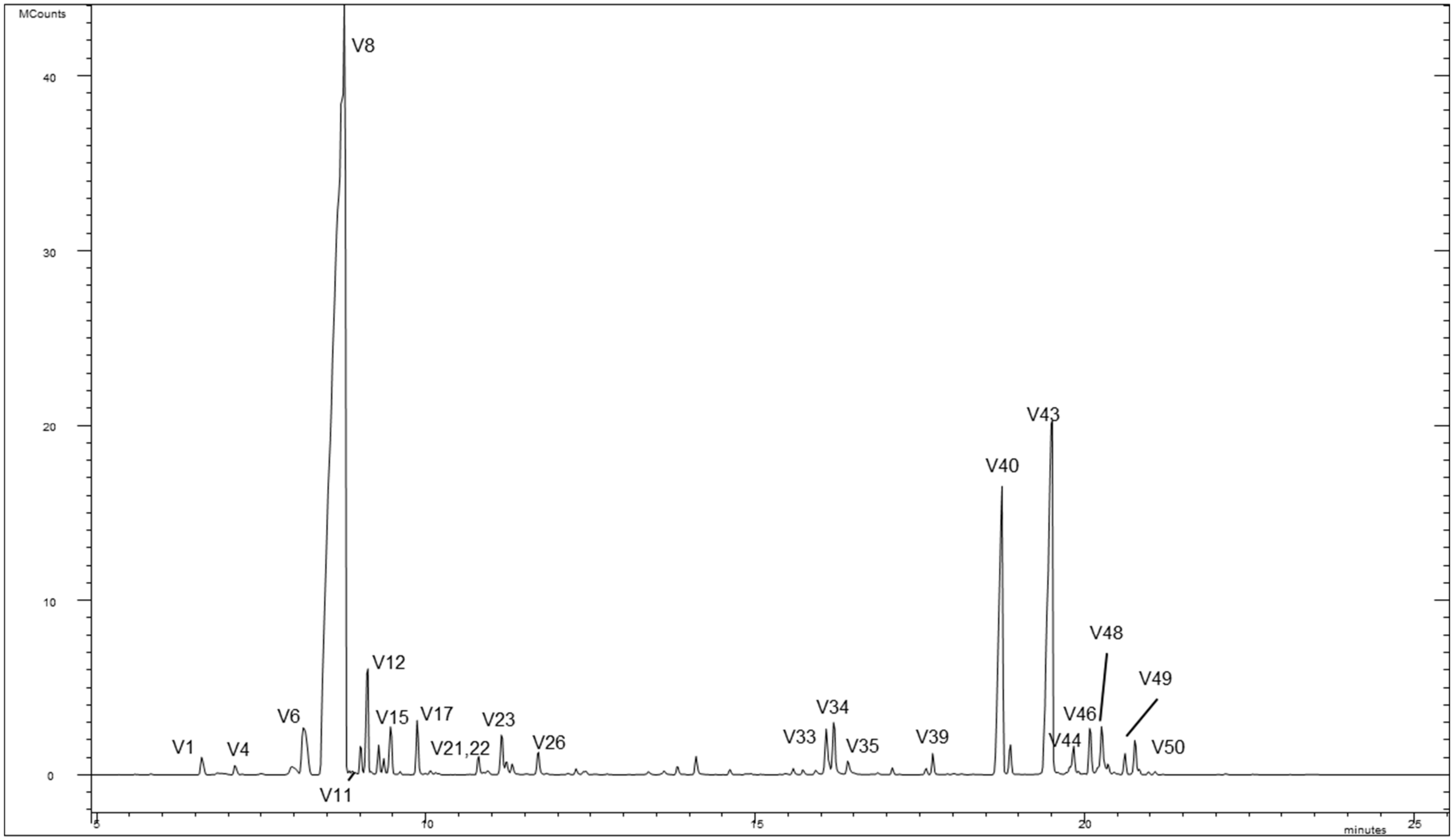
MCounts
40
30
20
10
0
V8
V1
V4
V6
V11
V12
V15
V17
V21,22
V23
V26
V33
V34
V35
V39
V40
V43
V44
V46
V48
V49
V50
5
10
15
20
25
minutes

S2 (m)

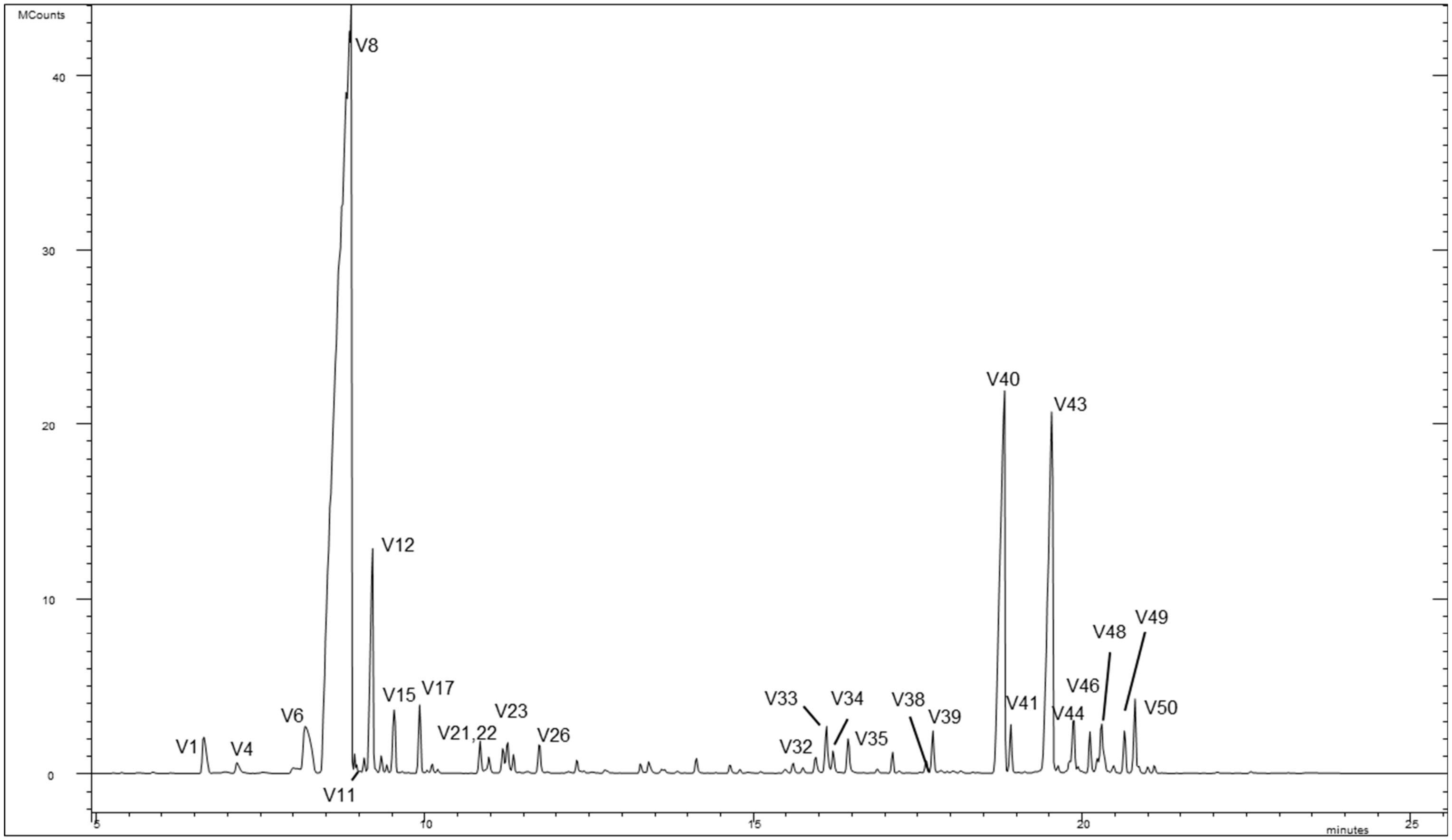

MCounts
40
30
20
10
0
5
10
15
20
25
minutes
V1
V4
V6
V8
V11
V12
V15
V17
V21,22
V23
V26
V32
V33
V34
V35
V38
V39
V40
V41
V43
V44
V46
V48
V49
V50

S2 (n)

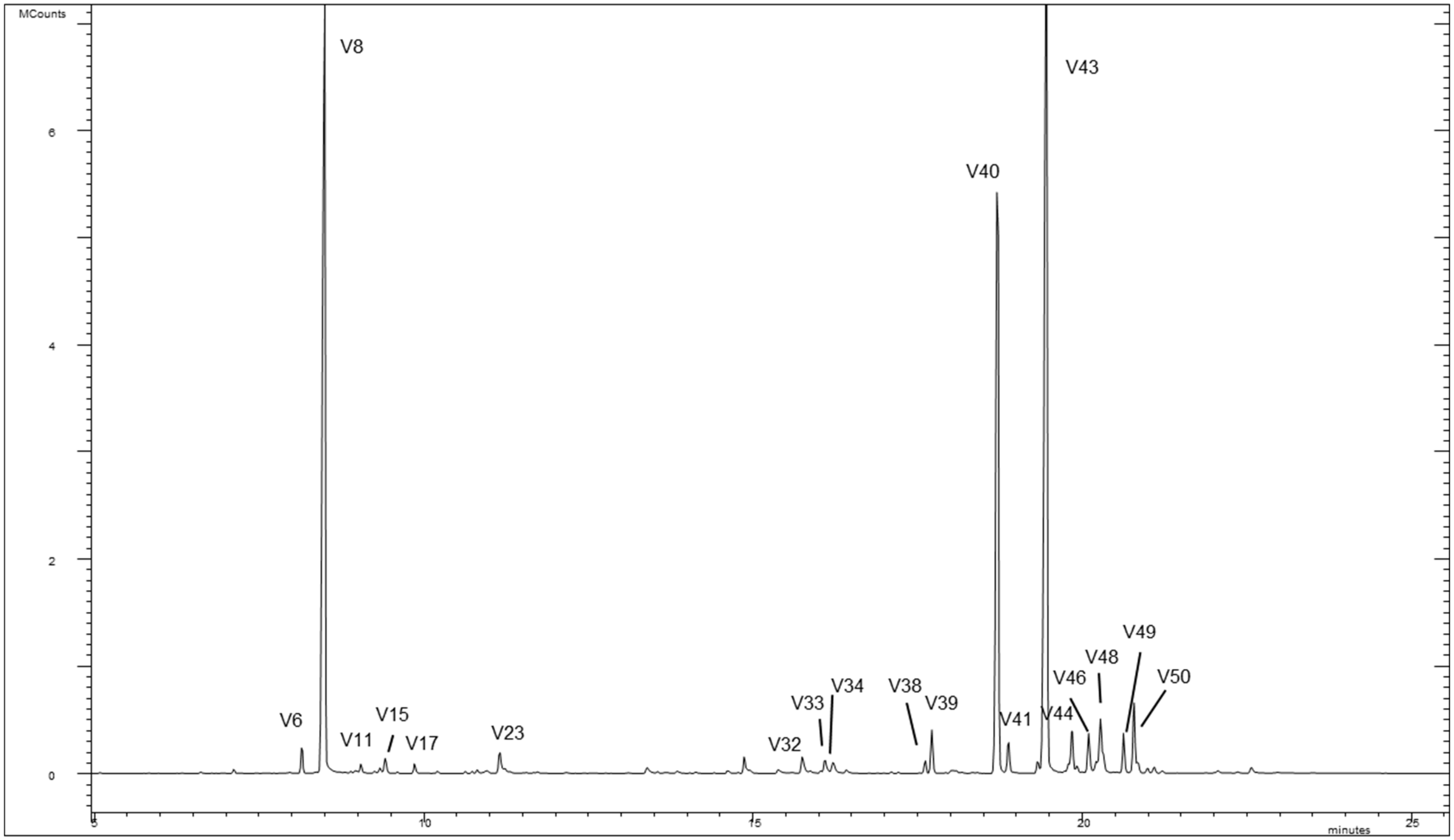
MCounts
6
4
2
0
5
10
15
20
25
minutes
V6
V8
V11
V15
V17
V23
V32
V33
V34
V38
V39
V40
V41
V43
V44
V46
V48
V49
V50

S2 (o)

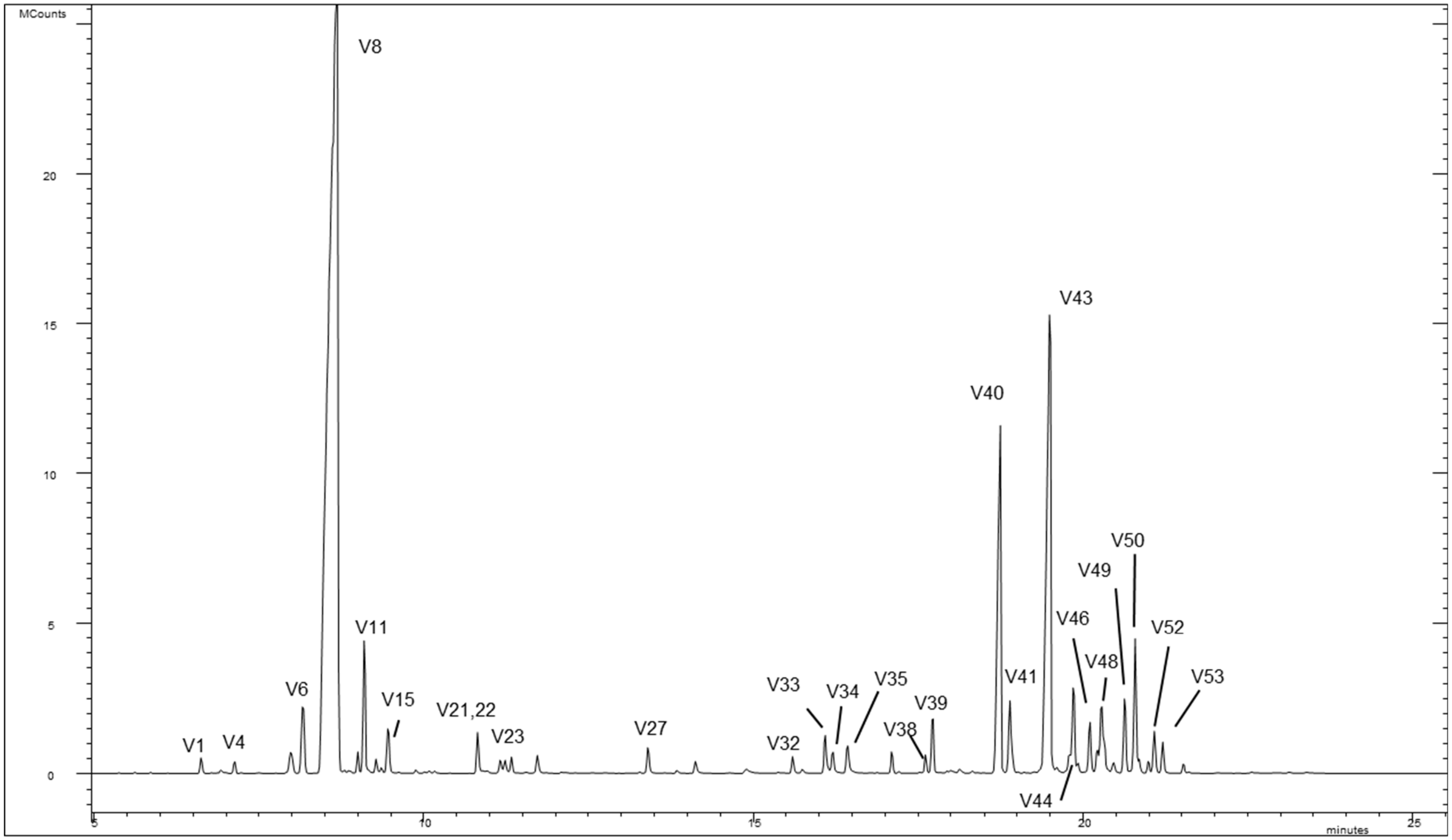

MCounts
20
15
10
5
0
5
10
15
20
25
minutes
V8
V43
V40
V50
V49
V46
V52
V11
V48
V53
V41
V6
V15
V33
V34
V35
V39
V21,22
V38
V27
V1
V4
V23
V32
V44
